# Emerging Role of ctDNA Fragmentomics and Epigenetic Signatures in the Early Detection, Minimal Residual Disease Assessment, and Precision Monitoring of Renal Cell Carcinoma

**DOI:** 10.1111/jcmm.71019

**Published:** 2026-01-30

**Authors:** Hossam Kamli, Najeeb Ullah Khan

**Affiliations:** ^1^ Department of Clinical Laboratory Sciences College of Applied Medical Sciences, King Khalid University Abha Saudi Arabia; ^2^ Institute of Biotechnology and Genetic Engineering The University of Agriculture Peshawar Pakistan

**Keywords:** ctDNA, early detection, epigenetic signatures, fragmentomics, minimal residual disease, precision monitoring, renal cell carcinoma

## Abstract

Renal cell carcinoma (RCC) presents a significant global health challenge, with a substantial proportion of patients diagnosed with advanced or metastatic disease due to the limitations of current diagnostic imaging and the lack of validated non‐invasive biomarkers. These conventional methods, including computed tomography and magnetic resonance imaging, often lack the sensitivity and specificity to differentiate benign from malignant small renal masses reliably or to detect minimal residual disease (MRD) post‐treatment. This review explores the transformative potential of liquid biopsy, explicitly focusing on circulating tumour DNA (ctDNA) fragmentomics and epigenetic signatures, to overcome these clinical hurdles. This review also explores how the analysis of ctDNA fragmentation patterns—such as size distribution, end motifs, and nucleosome footprints—provides a mutation‐independent method to enhance RCC detection, even in low‐shedding tumours. Concurrently, RCC‐specific epigenetic alterations, particularly DNA methylation profiles, offer particular biomarkers for early detection, tumour classification, and prognostication. This Review examines evidence that integrating these multi‐analyte approaches—combining fragmentomic and epigenetic data—synergistically improves diagnostic accuracy, enables sensitive MRD assessment, and allows precision monitoring of treatment response and tumour evolution. Despite existing technical and biological challenges, the convergence of ctDNA fragmentomics and epigenetic profiling heralds a new era for the non‐invasive, dynamic, and personalised management of RCC, promising to improve patient outcomes through earlier intervention and tailored therapeutic strategies.

## 1 Introduction

Renal cell carcinoma (RCC) provides a mounting global public health burden, with around 431,000 new cases and ~180,000 deaths worldwide as of 2020; about 2.4% of all adult cancer diagnoses [1]. The SG‐prevalent rates are particularly standardised incidence rate (ASIR) of ~6.1 per 100,000 in males and ~3.2 per 100,000 in females, compared to overall mortality, evincing substantial geographic heterogeneity still [2] GLOBOCAN data discussed in Epidemiology and Prevention of RCC. Early detection of small renal masses (SRMs) remains challenging, as benign or indolent lesions are often overtreated or misclassified (Liquid Biopsy as a New Tool for Diagnosis and Monitoring in RCC; Comprehensive Characterisation of Cell‐free Tumour DNA in Plasma and Urine). Traditional diagnostic tools that include contrast‐enhanced Computed Tomography (CT), Magnetic Resonance Imaging (MRI), or ultrasound have reasonable accuracy in the setting of larger or higher invasive RCC [3], but provide neither sensitivity nor specificity to differentiate benign from malignant small renal lesions, and do not lend themselves easily to frequent surveillance or evaluating minimal residual disease because of invasiveness or cost [4, 5]. Non‐invasive biomarkers like ctDNA, epigenetic changes, and fragmentomics can improve early diagnosis, treatment, and prognosis in RCC.

A liquid biopsy is the detection and analysis of tumour‐derived material, including ctDNA, cell‐free DNA (cfDNA), circulating tumour cells (CTCs), and extracellular vesicles in bodily fluids, as an alternative to tissue biopsy—a minimally invasive, dynamic approach [6]. Its benefits include tracking tumour heterogeneity and temporal evolution, identifying actionable genomic and epigenomic alterations, evaluating treatment response, and incorporating Minimal Residual Disease (MRD) monitoring across various cancer types. In early‐stage cancers, ctDNA, methylation, and CTCs show substantial diagnostic and predictive value, supporting the liquid biopsy's clinical utility [7]. In invasive cancer, notably RCC, limited detection is reported at stages when the majority of patients present [8], yet performances including combined fragmentomics assayed with cfDNA and machine learning (ML) have achieved areas under the curve of AUCs of ~0.966 (validation) and ~0.952 (external cohort—notably high sensitivity plus specificity for early‐stage disease) Glennon et al. [9]. An RCC‐specific NGS panel detected cfDNA and extracellular vesicle DNA mutations, showing the liquid biopsy's growing clinical relevance for diagnosis, prognosis, and monitoring in RCC [10].

Circulating tumour DNA (ctDNA) is composed of small fragments of the DNA that are released into the bloodstream (and on occasion other body fluids) of a patient from cells within a tumour by mechanisms including apoptosis, necrosis, or active secretion, and comprises a subset of total cfDNA [2, 5]. Biologically, a web of factors influences the quantity and fragment‐size distribution of ctDNA, including tumour mass, vascularity, cellular turnover, and clearance from circulation, all of which contribute to detection sensitivity and can be prohibitive in early‐stage disease. To date, ctDNA has been successfully implemented in various cancer types to perform mutation profiling, identify therapy resistance, prognosticate or monitor treatment response, and assess MRD [11]. For instance, in lung, colorectal, and breast cancers, ultra‐deep sequencing or digital Polymerase Chain Reaction (PCR) of ctDNA can identify somatic mutations with variant allele fractions as low as ~0.1% or less, earlier than by imaging in some settings, to detect progression. In RCC, ctDNA is challenging to detect due to low levels, background cfDNA, assay limits, and tumour heterogeneity [12]. For RCC, untargeted ctDNA sequencing identifies A minority of patients, ~27.5% in plasma [13], and even more personalised and sensitive methods can detect only ~50% in rural and even locoregional disease, which is still not strong enough for clinical use [14].

Fragment OMICS is the study of properties of cfDNA and ctDNA beyond sequence mutations, such as median fragment size distribution, chromosome 1 compendium/copy number variation patterns, and stalk‐to‐stalk regional coverage biases. For instance, in RCC, a survey in P53 clear cell RCC patients observed that the presence of detectable ctDNA was significantly associated with an increased small fragment (50–166 bp) to large fragments (167–250 bp) ratio and showed that fragment size, as well as mutant allele frequency, could be correlated with the cancer‐specific survival [15]. More recently, the DECIPHER‐RCC assay integrating Copy Number Variation (CNV), fragment size distribution and nucleosome footprint has demonstrated remarkable diagnostic accuracy (AUC 0.966 in a validation cohort; sensitivity ~90.5%, specificity ~93.8%), as well as consistent performance when applied to an external cohort of patients with RCC using another reference genome (area under the curve AUC≈0.952), regardless of tumour stage and histologic subtype [7]. This work also demonstrated effective differentiation of benign and malignant renal masses with these fragmentomic features. Recent technical developments in fragmentomic analysis include development of standardised pipelines to minimise biases introduced by library prep and features, low‐pass whole genome sequencing for comprehensive fragmentomic profiling, ensemble ML approaches to integrate multiple fragmentomic modalities, and *in silico* size‐selection to enrich tumour‐derived fragment signals.

Epigenetic alterations in ctDNA, particularly DNA methylation and chromatin accessibility, have been identified as promising biomarkers for cancer detection, tissue‐of‐origin assignment, and tumour classification, including RCC. DNA methylation is a stable, robust, and tissue‐specific epigenetic mark that can serve as an accurate means of classifying tumour origin. A pioneer study of Semaan et al. [16] reported on RCC molecular subtypes which fall into two main epigenetic groups (C1 [clear cell, papillary, translocation RCC] and C2 [chromophobe RCC, oncocytoma]), with the C1 tumours displaying 3‐fold more hypermethylation than the C2 group, particularly within gene bodies and intergenic stretches of DNA that are associated with more aggressive tumour behaviour and an adverse clinical prognosis. Similarly, Hauduc et al. [17] had identified 864 differentially methylated CpG islands when comparing RCC tissue to normal kidney, of which 96.3% showed hypermethylation; they constructed a 23‐marker methylation signature based on the methylation status, which yielded an AUC of 0.999 in tissue and 0.852 (plasma) as the signature for early detection of RCC. In addition to methylation, Assay for Transposase‐Accessible Chromatin using sequencing (ATAC‐seq) has revealed differential accessibility profiles in RCC tumour cells, including subtype‐specific regions at genes such as CA9, KRT14, RADIL, and PDGFRA, which further characterise epigenetic heterogeneity within and between tumours [18]. Notably, cfDNA methylation profiling with cfMeDIP‐seq has also been beneficial in identifying sarcomatoid differentiation in RCC—a highly aggressive subtype—yielding an AUROC of 0.95 with a sarcomatoid methylation score [16].

The fragmentomic and epigenetic cfDNA‐based analysis in ctDNA assays shows excellent potential to improve the management of RCC by comprehensively understanding and leveraging the interactions among multiple ctDNA features. Fragmentomics provides information on DNA fragment size, nucleosome positioning, and fragmentation patterns, thereby improving the sensitivity of low‐tumour‐burden detection. In contrast, epigenetic markers such as DNA methylation and chromatin accessibility lend tissue‐specific and regulatory insights that better classify tumours and infer their origin [16, 19]. This multimodal strategy addresses a significant void in RCC early detection and MRD monitoring, specifically the suboptimal sensitivity of mutation‐based ctDNA assays due to low mutation levels and the heterogeneity characteristic of RCC [20, 21]. In addition, integrating fragmentomic and epigenetic information could promote personalised strategies for monitoring therapeutic response, potentially leading to earlier therapeutic interventions and improved outcomes [18, 22]. These complementary ctDNA features can be combined to yield a more comprehensive, non‐invasive, and molecularly personalised biomarker platform suited to the distinct molecular characteristics of each patient's RCC.

## 2 Renal Cell Carcinoma: Clinical and Molecular Overview

Renal cell carcinoma (RCC) is a molecularly diverse tumour that can be categorised into separate histological subtypes, including clear‐cell RCC (ccRCC) and other less prevalent types, such as papillary RCC and chromophobe RCC, based on genomic mutations, transcriptomic programs, clinical outcomes, and therapeutic responses. ccRCC, for instance, is characterised by frequent loss of the VHL gene on chromosome 3p in addition to mutations in chromatin remodelling genes, including *PBRM1*, *BAP1*, and *SETD2*, which impact tumour biology, e.g., metabolic reprogramming, hypoxia signalling, as well as clinical aggressiveness and prognosis [23, 24]. These molecular insights have been utilised in a number of recent clinical trials to enhance therapy: ICI combined with anti‐angiogenic agents, targeting VEGF signalling, has been shown in meta‐analyses and systematic reviews for improvement in OS, PFS, and ORR relative to monotherapy for RCC without markedly increasing high‐grade adverse events over many comparisons [25]. Taken together, molecular classification, mutation profiling, and biomarker development collectively frame a transition to an era of therapy in RCC based on the matching of histologic subtype, mutation status, expression signatures, and immune microenvironment with the optimal therapeutic option.

### 2.1 RCC Subtypes: Molecular and Genetic Landscape

RCC is a heterogeneous group of malignancies characterised by several molecular, genetic, and clinical features that influence the management of these tumours. The most frequent type, ccRCC—constituting approximately 70%–75% of cases—is defined by VHL loss and HIF pathway activation, in association with recurrent mutations in chromatin‐modifying genes, which contribute to the susceptibility of this disease to VEGF‐targeted therapies and immunotherapy. Papillary RCC (pRCC) is the second most prevalent subtype, which is further classified into Type 1 and Type 2. Type 1 is often driven by MET activation, and Type 2 can present with metabolic alterations, such as FH loss and the CpG island methylator phenotype. ChRCC (constituting nearly 5%) is genetically distinct and manifests with whole‐chromosome losses, as well as mutations in TP53 and genes related to the mTOR pathway. Despite the fact that these subtypes (collecting duct carcinoma [CDC], MiT family translocation RCC [tRCC], succinate dehydrogenase [SDH]‐deficient RCC, and hereditary leiomyomatosis‐associated RCC [HLRCC]) are less common, they all have specific genetic drivers based on their histology and therapeutic implications that vary from platinum‐based chemotherapy in CDC to trials with targeted agents for hereditary syndromes. These differences highlight the importance of incorporating molecular characterisation into diagnosis, prognosis, and therapeutic strategies. An extensive summary of the molecular and genetic characteristics of RCC subtypes, such as incidence, genomic drivers, and clinical relevance, is presented in Table 1.

**TABLE 1:** Comprehensive molecular and genetic landscape of renal cell carcinoma (RCC) subtypes, their prevalence, key alterations, and clinical/therapeutic implications.

RCC subtype	Prevalence (reported)	Key molecular/genetic features	Clinical/therapeutic implications	References
Clear‐cell RCC (ccRCC)	~70%–75% (TCGA, reviews)	VHL inactivation (3p loss); HIF stabilisation; VEGF upregulation; mutations in PBRM1, BAP1, SETD2, KDM5C; CNAs (3p loss, 5q gain); high intratumour heterogeneity	Responsive to VEGF‐targeted therapy; BAP1 → poor prognosis; PBRM1 may predict ICI response; chromatin/metabolic alterations influence outcomes	[26, 27]
Papillary RCC (pRCC) – Type 1	~5%–10% of RCC (subset of pRCC)	MET activation (mutations, amplifications, chr 7 gains); chr 7 and 17 gains are common	MET inhibitors are effective in MET‐driven tumours; moderate sensitivity to VEGF/mTOR inhibitors	[28, 29]
Papillary RCC (pRCC) – Type 2	~10% of RCC (subset of pRCC)	Heterogeneous; CDKN2A loss; chromatin modifier mutations; FH loss (HLRCC); CIMP phenotype	FH‐deficient → aggressive, metabolic vulnerabilities; variable response to VEGF/mTOR therapy	[30, 31]
Chromophobe RCC (chRCC)	~5% of RCC	Whole‐chromosome losses; TP53, PTEN mutations; mTOR pathway (MTOR, TSC1/2); mitochondrial DNA and TERT alterations	Generally indolent; aggressive forms enriched for mTOR/TP53; mTOR inhibitors are a potential option	[23]
Collecting Duct Carcinoma (CDC)	< 1%–2% (rare; ~0.2% in single‐institution reports)	NF2, SMARCB1, CDKN2A alterations; MAPK & PI3K/AKT/mTOR activation; immune infiltration	Aggressive; platinum‐based regimens; immunotherapy combinations under study	[24]
MiT family translocation RCC (tRCC)	~1%–4% (more common in young patients)	TFE3/TFEB gene fusions (Xp11.2); MiT factor dysregulation	Aggressive; limited VEGF‐TKI efficacy; some benefit with ICIs or mTOR inhibitors	[32, 33]
Succinate dehydrogenase (SDH)‐deficient RCC	< 1% (very rare)	Germline SDHB/SDHC/SDHD mutations; succinate accumulation; epigenetic dysregulation	Often aggressive; hereditary syndromic associations; metabolic vulnerabilities	[23, 34]
Hereditary Leiomyomatosis & RCC (HLRCC)	Rare (< 1% of RCC; subset of pRCC2)	Germline FH mutations; fumarate accumulation; CIMP phenotype	Highly aggressive; requires genetic counselling; trial enrollment encouraged	[35]
Unclassified RCC	~4%–6%	Heterogeneous group; mixed or novel alterations (ALK fusions, chromatin/mTOR mutations)	Management is individualised; some fusions are targetable; trial/basket study enrollment is underway	[36]

#### 2.1.1 Driver Mutations and Pathway Alterations

##### 2.1.1.1 Major Subtype–Defining Drivers

RCC is defined by 3p loss in conjunction with biallelic inactivation of the VHL tumour suppressor, leading to HIF stabilisation and a dominant angiogenic programme that underpins the pivotal role of VEGF‐targeted therapies in this molecular subtype. Systematic multi‐platform, large‐scale profiling by the Cancer Genome Atlas measured these patterns and uncovered recurrent co‐mutations of chromatin regulators (in particular PBRM1, BAP1, SETD2) and commonly observed copy‐number alterations (notably 3p loss, 5q gain), which together define tumour transcriptional states, metabolism, prognosis, and immune micro‐environment profiles [37, 38]. Papillary RCC (pRCC) molecularly segregates across at least two biologic classes: Type 1, enriched for MET activating mutations/amplifications and chromosome 7/17 gains; and Type 2, a more diverse group including tumours with CDKN2A silencing and mutations in chromatin modifiers as well as specific hereditary HLRCC cases harbouring FH loss associated with accumulation of fumarate on an oncometabolite basis; these differences confer differential sensitivity to MET inhibitors, and motivate distinct trial designs/treatments based upon metabolic/epigenetic manipulation among Type 2 disease agents [39]. chRCC is defined by widespread whole chromosome loss (aneuploidy), a relatively low point mutation burden but recurrent TP53 and PTEN alterations, and, in aggressive subgroups, mutations in the mTOR pathway gene members, indicating potential mTOR‐targeted therapies for sensitive patients [40].

##### 2.1.1.2 Rare Hereditary and Fusion‐Driven Entities

Mendelian syndromes and rare, fusion‐driven tumours have unique, targetable biology and significant clinical implications. Hereditary leiomyomatosis and renal cell carcinoma (HLRCC) is caused by germline mutations in the FH gene, resulting in fumarate accumulation, widespread epigenetic changes (CIMP‐like methylation), and clinically aggressive disease, necessitating genetic counselling and specific surveillance therapy strategies [41]. Succinate dehydrogenase (SDH)‐deficient RCC is a rare germline‐related neoplasm caused by the inactivation of SDHx genes with succinate accumulation and distinctive morphologic and clinical features [42]. MiT family refers to the microphthalmia (MiT) family of transcription factors, which translocate renal cell carcinomas (RCCs) harbouring chimeric transcription factors, known as TFE3/TFEB fusions, that tend to occur in younger individuals and exhibit transcriptional programs different from VHL‐driven tumours, often requiring fusion‐directed diagnostic tests (FISH/RNA‐seq) for appropriate clinical management Liu et al. [43]. CDC is a rare tumour with unique genomic and immune characteristics and an aggressive behaviour that usually requires treatment with platinum‐based regimens or enrollment in trials of immune and targeted combinations [44].

##### 2.1.1.3 Cross‐Cutting Pathways and Clinical Implications

Common themes of human RCT have emerged across subtypes, including recurrent pathway alterations in oxygen sensing/HIF–VEGF angiogenesis (ccRCC predominant), chromatin and epigenetics dysregulation (PBRM1, BAP1, SETD2), receptor tyrosine kinase signalling (MET in pRCC and rare kinase fusions in unclassified tumours), PI3K–AKT–mTOR activation (selected chRCC and dedifferentiated cases), and metabolic enzyme dysfunction generating oncometabolites with wide‐ranging epigenomic consequences for gene regulation [23]. Clinically, such molecular maps inform Diagnosis, whether of diagnostic classification [24], germline testing when appropriate, and therapeutic matching—e.g., VEGF‐TKIs/HIF‐2α inhibitors for angiogeniclear ccRCC; MET inhibitors for MET‐driven pRCC; mTOR inhibitors in mTOR/TSC‐aberrant cancers and metabolic/epigenetic strategies or clinical trials for FH/SDH‐deficient cancers. Consequently, the integration of panel‐based sequencing (including fusion RNA assays), copy‐number and methylation profiling, where appropriate, is critical to both contemporary RCC management and trial design [32, 35].

### 2.2 Current Diagnostic Modalities and Their Limitations

#### 2.2.1 Imaging Techniques and Tissue Biopsies

Cross‐sectional imaging with contrast‐enhanced CT and MRI continues to be the foundation for RCC Diagnosis, staging, and preoperative preparation; CT has excellent spatial resolution to evaluate tumour size, presence of thrombus within the renal vein/inferior vena cava (IVC), and metastatic workup while MRI provides superior soft‐tissue contrast often being favoured when iodinated contrast agents are contraindicated or vascular/IVC assessment is desired [45, 46]. Meta‐analyses indicate that CT and MRI show good sensitivity but limited specificity for distinguishing malignant from benign renal masses. FDG‐PET/CT has limited utility for primary renal tumour characterisation due to physiologic tracer excretion and variable tumour uptake, but it demonstrates reasonable performance for detecting metastatic disease and may be helpful as an adjunct in equivocal staging or therapy response assessment [47, 48].

Image‐guided percutaneous renal mass biopsy is increasingly used to obtain histologic confirmation of indeterminate renal lesions [49]. Systematic reviews report a modest rate of non‐diagnostic results, with repeat biopsy frequently providing diagnostic clarification [49, 50]. Institutional series further demonstrate high technical success and diagnostic yield, although performance varies by lesion size, operator experience, and biopsy technique [49]. Limitations include sampling error in heterogeneous tumours, potential tumour undergrading, procedure‐related complications, and limited capacity for comprehensive molecular profiling from small tissue cores [51].

#### 2.2.2 Biomarkers in Clinical Use and Drawbacks

To date, no blood or urine biomarker has been validated for routine use in the diagnosis of RCC. Several potential tissue and circulating markers are being investigated. The HIF‐regulated protein carbonic anhydrase IX (CAIX) is highly expressed in clear‐cell RCC and has been assessed as a prognostic and predictive marker in retrospective analyses and trial correlative studies, but is not generally recommended as a stand‐alone testing marker due to limited specificity across contexts [52, 53]. ctDNA and additional liquid‐biopsy analytes such as cfDNA, circulating tumour cells, or exosomes are promising for noninvasive molecular profiling and disease monitoring yet with lower detection rates in RCC compared to many other solid tumours; reported ctDNA detection rates vary widely despite being affected by the level of latency to volume associated with the tumour burden, stage (roughly 17%–54% in tumour‐guided studies), sequencing depth and number of target genes assayed [54, 55]. These analytical and biological constraints—namely, the low ctDNA fraction in most localised RCCs, the lack of assay standardisation, and varying sensitivity across subtypes—currently preclude the routine clinical implementation of ctDNA in early diagnostic or complete molecular stratification [56].

#### 2.2.3 Emerging Diagnostic Approaches: Radiogenomics and Artificial Intelligence

Radiogenomics and artificial intelligence (AI) based radiomics aim to integrate imaging with molecular biology by quantifying imaging features that correlate with histology, grade, driver mutations, and outcome. A number of smaller retrospective studies and cohorts, on the other hand, demonstrate the promising capacity of radiomic signatures and machine‐learning models to differentiate between ccRCC and non‐ccRCC, predict histological Fuhrman/WHO grade, and infer mutational status, with AUCs typically ranging from 0.75 to 0.90 in single‐center studies [57]. Nevertheless, multi‐center harmonisation, batch effect, standardised imaging protocols, external validation, and prospective clinical trials are lacking; these technical and reproducibility challenges have impeded clinical translation to date [58]. Radiogenomic approaches, combined with AI‐augmented image interpretation, may decrease reliance on invasive biopsy for specific indications and enhance non‐invasive subtyping; however, rigorous prospective validation and establishment of regulatory pathways are necessary before the approach can be considered for routine clinical use [48, 59].

### 2.3 Unmet Clinical Needs in RCC Management

#### 2.3.1 Diagnostic Sensitivity and Specificity Gaps

While cross‐sectional imaging is crucial for staging and surgical planning, it lacks the practicality of making a firm distinction between benign and malignant renal masses and RCC subtypes. Forthcoming Comparative studies and meta‐analyses Large comparative studies and meta‐analyses have noted high sensitivity for detection of renal malignancy at a cost of variable to occasionally low specificity by lesion type and imaging protocol, yielding clinically meaningful false positives (e.g., oncocytoma misclassified as RCC) and false negatives for small or hypovascular tumours [60]. Machine‐learning radiomic models increase discrimination in single‐center series—such as a CT‐based radiologic‐radiomic model that achieved ~90% sensitivity and ~89% specificity for distinguishing clear‐cell RCC from other renal masses in cross‐validation [61]—but promising results are still limited by single‐center design, heterogeneous imaging protocols, and external validation [62, 63]. Image‐guided percutaneous biopsy improves certainty of diagnosis, yet remains fallible; pooled analyses report nondiagnostic/core failure rates approximately 10%–15% (overall pooled nondiagnostic ≈14.1%), repeat biopsy converts many to diagnostic in the majority of cases, and sampling error is a key limitation with intratumour heterogeneity [64, 65]. PET imaging is most useful for metastatic evaluation rather than primary renal lesion characterisation, with performance varying by tracer and tumour histology [66, 67]. Collectively, these results, as mentioned in Table 2, demonstrate that neither imaging nor biopsies were completely diagnostic or molecularly informative for most patients, and that there is an urgent demand for validated non‐invasive classifiers with cross‐center generalisation.

**TABLE 2:** Diagnostic performance and limitations of current RCC modalities.

Modality	Metric	Performance/range	Notes and limitations	Key references
CT/MRI	Sensitivity for renal malignancy	High (> 90% in most series)	Excellent for detection; poor specificity for subtype; oncocytomas often misclassified	[68]
	Specificity	Variable (50%–80%)	Limited discrimination of benign vs. malignant, especially < 4 cm masses	[48]
Radiomics/AI (CT‐based)	Sensitivity	~90%	Performance comparable to expert radiologists in single‐center models	[69]
	Specificity	~89%	Lacks external validation; heterogeneity in imaging protocols	[70, 71]
Percutaneous biopsy	Diagnostic yield	85%–90%	Nondiagnostic in ~10%–15% of cases; repeat biopsy often successful	[72]
	Nondiagnostic rate	Pooled 14.1%	Sampling error due to tumour heterogeneity	[53]
PET/PET‐CT	Primary RCC lesion detection	Sensitivity ~50%–70%, specificity ~60%–80% (FDG)	Limited role for primary diagnosis; low uptake in ccRCC	[73]
	Metastatic detection	Sensitivity/specificity is higher (~80%–90%) with PSMA‐targeting tracers	Still investigational for RCC	[74]
ctDNA/Liquid biopsy	Detection (localised RCC)	~20%–30% (tumour‐guided VHL variant assays); ~50% with tumour‐informed personalised panels	Limited sensitivity in localised disease	[50, 75]
	Detection (advanced RCC)	50%–70%+	Higher detection rates in the metastatic setting	[13]
	Prognostic performance	ctDNA positivity predicted relapse: sensitivity 84%, PPV 90%	Tumour‐informed assays superior; prospective validation ongoing	[50]

#### 2.3.2 Necessity for Real‐Time, Non‐Invasive Monitoring Methods

There is an unmet need for real‐time minimally invasive technologies to detect recurrence earlier, monitor on‐treatment, and detect the development of resistant disease. Liquid biopsy (including ctDNA, cfDNA, circulating tumour cells, methylation assays, and exosomal biomarkers) has shown promise, but results have been inconsistent in RCC. Systematic reviews suggest that ctDNA levels are generally low in RCC compared to many other cancer types, and detection rates vary between methods, depending on assay type, tumour stage, and whether the approach is tumour‐informed or agnostic [13, 76]. Personalised tumour‐informed ctDNA assays show substantially higher sensitivity than untargeted panels, enabling more frequent ctDNA detection in localised disease and providing substantial prognostic value for relapse, whereas tumour‐agnostic approaches report considerably lower detection rates [50, 77]. A proportion of studies find that ≥ 1 single driver variant (e.g., VHL) is detectable in plasma in ~20%–30% of cases, as reported above [75, 78]. Methylation‐based cfDNA approaches and ultra‐deep, tumour‐guided sequencing are among the most sensitive strategies for RCC.20. However, prospective trials have yet to demonstrate that outcomes improve with ctDNA‐guided decision‐making [13, 79]. Radiogenomic and AI techniques can also support liquid biopsy by enabling frequent, image‐based monitoring of phenotypes; however, their widespread adoption in the clinic requires multi‐center harmonisation, validation in prospective patient cohorts, and regulatory approval [51, 80]. Ultimately, validated integrated noninvasive platforms, high‐sensitivity ctDNA ± methylation assays, plus harmonised radiomics/AI would be of great value for real‐time monitoring of molecular and burden states—particularly for the early detection of recurrence and adaptive therapeutic modification.

## 3 Circulating Tumour DNA (ctDNA): Concepts and Technologies

Circulating tumour DNA (ctDNA) levels in RCC vary with stage, tumour burden, and assay type, being low in localised disease and higher in metastatic cases. Longitudinal analysis shows ctDNA reflects tumour evolution and emerging resistance. Tumour‐informed and methylation‐based assays improve sensitivity and can predict recurrence even in localised RCC. As outlined in Table 3, these results underscore the dichotomous nature of ctDNA in RCC: highly informative and clinically actionable in advanced disease when personalised with ultra‐sensitive strategies, increasingly favourable also in earlier stages, but ultimately limited by biological shedding variance, assay heterogeneity, and pre‐analytical confounders.

**TABLE 3:** ctDNA detection rates, assay characteristics, and clinical relevance in renal cell carcinoma (RCC).

Study (year)	Cohort/setting	Assay platform	Stage	Detection rate	LOD	VAF	Sample	Key notes
[81]	Systematic review (5–24 pts/study)	Mixed (NGS, PCR, methylation)	Mixed	17%–54%	Variable	Low in localised	Plasma/urine	Detection is highly assay‐dependent, with low sensitivity in early RCC
[75]	Multi‐cancer; RCC subset	Targeted NGS	Localised + met	Among the lowest in localised RCC (< 50%)	~0.1%–1%	Not reported	Plasma	RCC is confirmed as a low‐shedding tumour in the localised stage
[82]	Advanced RCC	Tumour‐agnostic NGS panels	Metastatic	79%	~0.5%–1%	Higher with burden	Plasma	Strong correlation with tumour burden (sum of diameters)
[83]	mRCC, longitudinal	73‐gene panel (CLIA)	Metastatic	Not pooled (serial detection focus)	0.1%–0.5%	Dynamic, ↑ with progression	Plasma	Captured clonal evolution, resistance variants in real time
[75]	Resected RCC (peri‐op)	Tumour‐informed mPCR‐NGS (Signatera)	Localised	50% pre‐op; post‐op+ prognostic	~0.01%	Larger tumours ctDNA+	Plasma	ctDNA+ predicted recurrence (Se 84%, PPV 90%)
[84]	mRCC (real‐world)	Guardant360 (70+ genes)	Metastatic	Frequent detection; variable	~0.1%	Variable: CHIP confounder	Plasma	Feasible in clinic; highlighted CHIP artefacts
[85]	RCC (localised + advanced)	Methylation/fragmentomics	Mixed	Higher sensitivity vs. mutation panels	~0.01% (tumour fraction)	NA	Plasma/urine	Improved detection in localised RCC; distinguishes RCC vs. controls

### 3.1 Advanced Technologies for ctDNA Detection and Analysis

Novel technologies for detecting ctDNA have gradually improved sensitivity and specificity and expanded applications across various cancers, including RCC. Conventional modalities, including ddPCR and BEAMing, enable ultra‐sensitive identification of known hotspot mutations but are less readily amenable to broader genomic coverage due to their limited multiplexing capacity. On the other hand, NGS‐based approaches (in particular, tumour‐informed designs that incorporate unique molecular identifiers) enable comprehensive analysis of single‐nucleotide variants, copy number alterations, fusions, and clonal evolution; however, they come at a higher cost and require more advanced bioinformatic expertise. Most recently, methylation‐based assays and fragmentomics have expanded the analysis of ctDNA beyond mutation detection (< 0.0001% VAF) and have also enhanced performance in low‐shedding tumours (such as RCC) by leveraging epigenetic and fragmentation features. Novel platforms, such as PhasED‐Seq, have achieved detection limits exceeding 0.95. Taken together, this wide variety of technical approaches (summarised in Table 4) illustrates the tension between sensitivity and both scope and feasibility, emphasising that assay selection must be closely paired to the clinical context.

**TABLE 4:** Comparative overview of advanced technologies for ctDNA detection and analysis: principles, analytical sensitivity, performance metrics, applications, strengths, and limitations across oncology and renal cell carcinoma research.

Technology	Principle/platform	Typical LOD (VAF/TF)	Sensitivity/specificity	Breadth of detection	Key applications	Strengths	Limitations/challenges	References
NGS (targeted panels, hybrid capture, tumour‐informed assays)	Parallel sequencing with UMIs and error suppression	~0.1%–1% (standard); ~0.01% (tumour‐informed, PhasED‐Seq)	High (70%–95% sens. in advanced); specificity > 95%	Broad (SNVs, indels, CNAs, fusions, MRD)	Genomic profiling, resistance detection, MRD	Broad coverage, scalable, tracks clonal evolution	Costly, bioinformatics‐heavy, and less sensitive in low‐shedding tumours	[75, 86]
ddPCR	DNA partitioned into droplets, probe‐based PCR	~0.01%–0.1% VAF	Very high for targeted (≥ 95% spec.)	Narrow (hotspot SNVs/indels)	Mutation hotspot detection, therapy monitoring	Ultra‐sensitive, low cost, fast turnaround	Low multiplexing cannot detect novel variants	[87]
Methylation assays (bisulfite PCR, cfMeDIP‐seq)	CpG methylation and fragmentation analysis	~0.01% TF	High (sens. > 90%, spec. > 95%)	Epigenome‐wide or targeted loci	Early detection, tumour typing, and RCC localised detection	Sensitive in early cancers, robust in low VAF	Sensitive to cfDNA handling, high computational load	[88, 89]
BEAMing	Beads + emulsion PCR + flow cytometry	~0.01% VAF	High (80%–90% sens., high spec.)	Focused (mutations only)	Early ctDNA detection, mutation quantification	Ultra‐sensitive, quantitative	Labor‐intensive, specialised equipment, low clinical uptake	[90, 91]
PhasED‐Seq	Phased variant sequencing with error suppression	~0.0001% VAF	Extremely high (detects < 0.001% AF); spec. > 99%	Mutation‐focused	MRD detection, relapse prediction	Ultra‐low LOD, detects minimal residual disease	Requires tumour tissue, limited to mutations, new tech	[92]
Fragmentomics/Nucleosome footprinting	Fragment length, end motifs, nucleosome positioning	Not VAF‐based (~0.5%–1% TF)	Moderate–high (AUC > 0.9 in some classifiers)	Genome‐wide fragmentation patterns	Early detection, tissue‐of‐origin, RCC urine/plasma	Mutation‐independent, orthogonal info	Experimental, non‐standardised, bioinformatics‐heavy	[19, 93]
Multi‐Omic platforms	Integrates mutations + methylation + fragmentomics + proteins via AI/ML	Variable; often < 0.01%	Very high (multi‐modal AUC > 0.95)	Pan‐omic, multi‐layered	Cancer screening, monitoring, and tumour classification	Maximises sensitivity and specificity; tumour‐type prediction	Expensive, complex, early validation stage	[94, 95]

### 3.2 Analytical and Biological Challenges in RCC ctDNA Analysis

The assessment of ctDNA in RCC is hindered by considerable analytical and biological challenges, especially in patients with early‐stage disease, where ctDNA concentrations may be minimal. Bettegowda et al. [75], have demonstrated that RCC is among the cancers with the lowest capacity to shed circulating tumour DNA (ctDNA), with detection rates in localised disease ranging from 17% to 54%. These findings were verified in systematic reviews across multiple cohorts [96]. Conversely, high detection rates are found in metastatic RCC, with Seyednasrollah et al. [97] reporting 79% ctDNA positivity in a cohort of 224 advanced RCC patients, which is directly proportional to tumour load. In localised RCC, radiation‐informed assays with improved sensitivity are more recent. For example, Groves et al. [98] identified ctDNA in 50% of preoperative plasma samples and demonstrated that both pre‐ and post‐nephrectomy ctDNA positivity were associated with recurrence (sensitivity, 84%; positive predictive value, 90%), supporting the prognostic utility of ctDNA despite a low detection rate.

Both tissues present significant challenges for detection, mainly due to biological factors. RCC tumours generally show little CM due to small tumour size, low vascularity, and distinctive patterns of cell death (necrosis, autophagy, or ferroptosis) that differ from apoptosis [93]. In addition, the low tumour mutational burden (TMB) in some RCC histologies decreases the likelihood of detecting informative variants at diluted tumour fractions and limits the applications of mutational assays [86]. To counteract these drawbacks, ultra‐high sensitivity approaches, including phased variant sequencing (PhasED‐Seq), methylation profiling, and fragmentomics, have been developed to detect ctDNA signals irrespective of the mutational burden [19, 88, 92].

Tumour heterogeneity and clonal evolution are other critical biological obstacles. McGranahan and Swanton [99] reported key evidence for substantial intratumoral heterogeneity in RCC, showing that mutations can be absent from one region despite being present elsewhere and thus hampering plasma‐based detection. Longitudinal studies in metastatic RCC using ctDNA have confirmed the dynamic selection of resistance clones under treatment pressure, demonstrating changes in variant allele frequencies representing clonal evolution [100]. This heterogeneity limits the sensitivity of single‐time‐point liquid biopsies, underscoring the importance of serial sampling, coupled with broad yet tumour‐informed assay designs.

**FIGURE 1:**
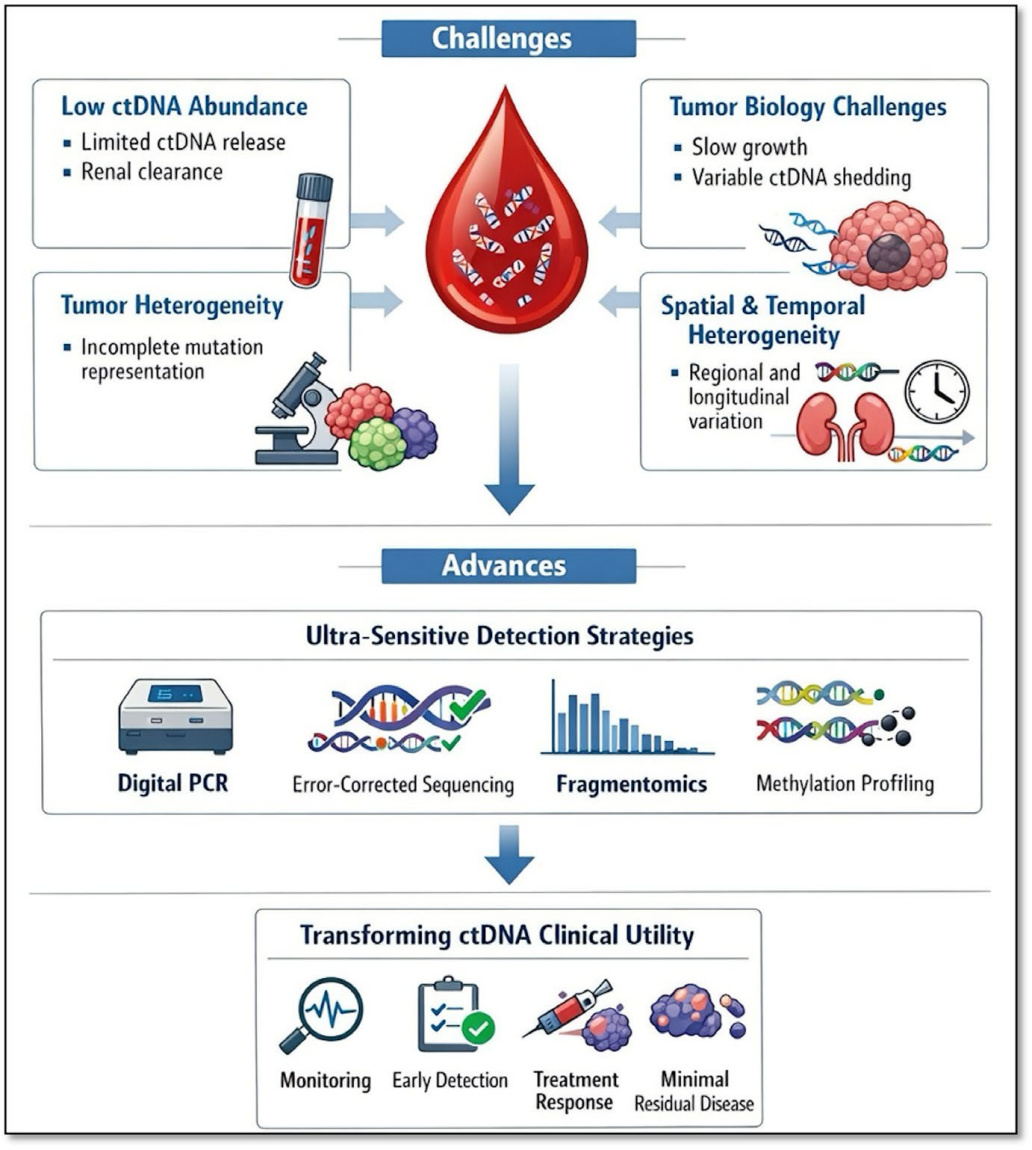
Analytical and biological challenges in circulating tumour DNA (ctDNA) analysis in RCC: low ctDNA abundance, tumour heterogeneity, clonal evolution, pre‐analytical confounders, and advanced detection strategies for improved sensitivity and clinical utility.

Alternatively, analytical confounding factors can complicate the interpretation of ctDNA. One of the most notable is clonal haematopoiesis of indeterminate potential (CHIP), which often harbours mutations in genes such as TP53, DNMT3A, and TET2. These CHIP‐associated variants can be incorrectly assigned as tumour‐derived ctDNA if paired white blood cell sequencing or bioinformatic filtering is not used [101, 102]. Pre‐analytical variability, such as delayed processing, improper tubes, or incorrect storage, can degrade cfDNA and reduce assay sensitivity by losing short tumour‐derived fragments [87, 89]. Key RCC ctDNA challenges—low tumour fraction, heterogeneity, and pre‐analytical factors—are shown in Figure 1. Metastatic disease yields stronger signals, while localised tumours need ultra‐sensitive assays, standardised protocols, and longitudinal sampling for clinical validation.

## 4 Translational Integration of ctDNA Fragmentomics

### 4.1 Principles and Biological Basis of Fragmentomics

Fragmentomics is the study of the fragmentation patterns of cfDNA, encompassing fragment size profiles, end motifs, and nucleosome potential. Tumour‐derived cfDNA, or ctDNA, has a shorter fragment length distribution (~90–150 bp) than that of cfDNA originating from healthy cells [93], which is around the size of nucleosomal packaging, about 166 bp. The exact boundaries of ctDNA fragments have unique motifs at the ends, which are also often enriched for specific nucleotide signatures at cutting sites, reflecting tumour‐specific nuclease activity [103]. Nucleosome footprints reflect tumour transcriptional activity and chromatin state, providing ctDNA information beyond mutations and serving as orthogonal signals for cancer detection and tissue‐of‐origin analysis [104].

### 4.2 Fragmentomics Enhancing Diagnostic Accuracy in RCC

In RCC, where the overall level of ctDNA is low, somatic mutation‐based assays are susceptible to sensitivity issues [75]. Fragmentomic features fill an essential gap as a complementary or secondary biomarker. Mouliere et al. [93] have demonstrated that analysing small‐fragment sizes enhances the detection of ctDNA across a range of cancer types, including RCC, by enriching for shorter tumour‐derived fragments. Similarly, Cristiano et al. [19] demonstrated that combining genome‐wide fragmentation profiles with machine learning enabled high classification performance in distinguishing between cancer patients and healthy controls, with AUC values exceeding 0.9. In RCC, a low‐shedding tumour, fragmentomic signals persist even when mutation‐derived ctDNA is undetectable. Fragmentation profiles correlate with stage, histology, and tumour burden, making fragmentomics a complementary tool to improve ctDNA assay sensitivity, specificity, and patient monitoring [93, 105].

### 4.3 Evidence From Clinical Studies in RCC Fragmentomics

Although still in its early stages, fragmentomics has shown promise in improving ctDNA detection in low‐shedding RCC. Mouliere et al. [93] reported that shorter DNA fragments (0.9 in pan‐cancer data sets) demonstrated feasibility in subsamples of RCC. Ulz et al. [105] demonstrated that nucleosome footprints derived from DNA fragments (< 150 bp) can provide gene expression and tissue‐of‐origin signals, facilitating biologically guided classification in RCC. Integrated cfDNA methylation and fragmentomics improve sensitivity compared with mutation‐only assays, supporting their complementary diagnostic and prognostic roles in RCC (see Table 5).

**TABLE 5:** Key clinical studies on ctDNA fragmentomics in RCC and related cohorts.

Study (first author, year)	Cohort/setting	Fragmentomic approach	Sample size	Key findings in RCC/low‐shedding tumours	References
Mouliere et al. 2018	Multi‐cancer, including the RCC subset	Fragment size selection (< 150 bp enrichment)	344 patients	Short‐fragment selection improved ctDNA detection; RCC showed increased sensitivity despite low ctDNA abundance	[93]
Cristiano et al. 2019	Pan‐cancer (20+ cancer types, incl. RCC)	Genome‐wide fragmentation + machine learning	236 patients	Achieved AUC > 0.9 for cancer detection; feasible detection in RCC patients with low‐shedding ctDNA	[19]
Ulz et al. 2016	Plasma cfDNA from cancer patients incl. RCC	Nucleosome footprinting (gene expression)	48 patients	cfDNA fragmentation reflected nucleosome occupancy; inferred expressed genes and tissue‐of‐origin, relevant to RCC detection	[105]
Shen et al. 2020	RCC and pan‐cancer cohorts	Integrated methylation + fragmentomics	~200 patients	Methylation‐fragmentomic signals improved detection sensitivity vs. mutation‐only; applicable in low‐ctDNA RCC cases	[106]
Liu et al. 2020	Multi‐cancer cohort with RCC subset	cfMeDIP‐seq + fragmentomic features	163 patients	Combined methylation and fragment length profiling enhanced classification and tumour‐type discrimination in RCC patients	[107]

### 4.4 Potential Clinical Applications and Future Directions

The clinical utility of ctDNA fragmentomics has been increasingly recognised in kidney cancer, particularly in early detection. In this area, mutation‐based assays have traditionally struggled due to the low abundance of circulating tumour DNA (ctDNA). Research by Cristiano et al. [19] demonstrated that machine learning using genome‐wide cfDNA fragmentation profiles achieved high diagnostic accuracy, with AUC values exceeding 0.9 in pan‐cancer settings. It is noteworthy that RCC, classified as low‐shedding based on mutation‐centric studies [108]—benefited from fragmentomics, highlighting the complementary nature of our strategy. Similarly, it was demonstrated that the selective enrichment of shorter cfDNA fragments (< 150 bp) improved sensitivity for ctDNA detection, even in RCC patients whose low‐VAF signals were undetectable by standard sequencing panels. These results indicate that fragmentomics may allow earlier diagnosis in localised RCC, for which current blood‐based diagnostic modalities are frequently insufficient.

Beyond early diagnosis, fragmentomics shows excellent potential for RCC‐MRD estimation and relapse prognosis. Post‐operative surveillance is an unmet clinical need, as early microscopic recurrence can be missed on radiographic imaging. Chidambaram et al. [109] demonstrated that the fragmentation patterns induced by nucleosomes can predict tissue‐specific transcriptional activity, providing a potential means of detecting RCC tissue‐specific recurrence signals in plasma. More recently, integrated approaches combining methylation and fragmentomic features, Wang et al. [110] have demonstrated enhanced sensitivity in MRD detection compared to mutation‐only assays. These approaches can detect molecular relapse months before radiological disease, thereby enabling earlier intervention or treatment intensification in high‐risk patients with RCC.

Long‐term follow‐up is another area in which fragmentomics could have a clinical impact. Dynamic alterations in cfDNA fragmentation profiles may serve as a novel marker to monitor changes in tumour burden and treatment response in patients, in addition to monitoring variant allele fraction. For example, Cristiano et al. [19] also noted that, although mutational collapse brought fragmentomic signals to levels lower than those easily detectable by standard approaches, visibility did not disappear entirely, providing the potential for continued monitoring in low shedding disease situations. As fragmentomics‐informed surveillance clinical trials develop, integration into the RCC management pathways could help personalise treatment escalation, including adjuvant immunotherapy decisions and detection of molecular progression before radiographic relapse. Taken together, fragmentomics offers a biologically rational, technically attainable, and clinically applicable method that may significantly enhance the value of ctDNA in precision oncology for RCC.

## 5 Epigenetic Signatures in ctDNA for RCC

Epigenetic changes, such as DNA methylation, histone modification, and chromatin remodelling, also play a crucial role in regulating gene expression without altering the underlying DNA sequence. In cancer, these changes can lead to the activation of oncogenes or the silencing of tumour suppressor genes, facilitating tumorigenesis and progression [111, 112]. DNA methylation, particularly in the CpG islands of promoter regions, is one of the most important mechanisms of gene silencing. Abnormal hypermethylation has been reported in several critical tumour suppressor genes in RCC, including VHL, RASSF1A, and P16INK4A [113]. Certain histone modifications, such as acetylation, methylation, and phosphorylation, also modulate chromatin structure and transcriptional expression levels, which are marked by histone modifications such as H3K4me3 and H3K27ac and are commonly associated with active transcription in RCC cells [114]. Chromatin‐remodelling complexes, such as SWI/SNF, regulate the positioning of nucleosomes and DNA accessibility; alterations in this function are associated with RCC progression and metastasis [115]. It may be feasible to measure these epigenetic changes in circulating tumour DNA (ctDNA), which could offer a minimally invasive test for early cancer detection, treatment response monitoring, and prognosis prediction [116].

New studies have discovered RCC‐specific epigenetic signatures in ctDNA as putative biomarkers. Differentially methylated regions (DMRs) have been identified in plasma circulating tumour DNA (ctDNA) of RCC patients, with high sensitivity and specificity for distinguishing between malignant and normal tissues [13, 117]. HYPAREA target: RASSF1A and APC promoter hypermethylation in ctDNA is associated with tumour stage, size, and aggressiveness [118]. In contrast to similar data in ctDNA, histone modifications also provide a window into tumour function, and H3K27ac and H3K4me3 enrichment have been found to mirror the transcriptional activation of oncogenic pathways in RCC [119]. Dysregulation of chromatin remodelling, particularly changes in SWI/SNF complex elements identified in circulating tumour DNA (ctDNA), was associated with worse outcomes and treatment resistance [120]. The incorporation of these epigenetic markers into ctDNA analysis may advance non‐invasive diagnosis and individualised, targeted treatment plans for RCC; however, validation in larger patient cohorts is necessary [121, 122].

### 5.1 Technologies for Epigenetic Profiling of ctDNA

Determination of epigenetic signatures from blood‐based circulating tumour DNA (ctDNA) has been recognised as a cornerstone in cancer diagnostics and monitoring. Bisulfite sequencing, methylation arrays, and enzymatic methylation assays are among the most widely used methods for profiling DNA methylation in circulating tumour DNA (ctDNA). Table 2 lists the primary technologies and their applications.

Bisulfite sequencing is the reference method in DNA methylation studies. This process involves treating DNA with sodium bisulfite, which converts unmethylated cytosines to uracils (which can be sequenced as thymines) without affecting methylated cytosines. They can be followed by next‐generation sequencing for mapping methylation patterns at single‐base resolution. Whole‐genome bisulfite sequencing (WGBS) yields genome‐wide methylation information, which is helpful for discovering new methylation‐based biomarkers in circulating tumour DNA (ctDNA). These target bisulfite sequencing panels can also narrow the focus to the regions of clinical interest, thereby minimising both cost and the need for restriction enzymes/probe‐based enzyme digestion and additional in‐depth sequencing analysis. Nevertheless, DNA is prone to degradation after bisulfite treatment, which might hamper its use in low‐input ctDNA sample analysis [123].

Methylation arrays, including the Illumina Infinium MethylationEPIC array, have been developed to assay methylation at thousands of predefined CpG sites across the genome in a high‐throughput manner. These arrays are relatively inexpensive and efficient for screening large numbers of individuals; however, they may fail to detect newly discovered or rare methylation events that were not part of the array's design. Nevertheless, they act as important tools for uncovering potential biomarkers and conducting large‐scale epidemiological investigations [124]. Arrays also permit comparison of multiple samples to perform population‐based analyses and to validate candidate methylation markers.

Enzymatic methylation assays, such as restriction enzyme real‐time PCR (REMT‐PCR) and their derivatives, or enzymatic conversion methods, are alternative methods to bisulfite treatment. These approaches may be more sensitive to DNA integrity and can have reduced input of DNA, which is particularly beneficial in the context of low amounts of circulating tumour DNA (ctDNA) in plasma. Approaches such as IMPRESS (Improved Methylation Profiling using Restriction Enzymes and smMIP sequencing), which integrate methylation‐sensitive restriction enzymes and single‐molecule molecular inversion probes, have been developed to enhance the detection of methylation changes [125]. Enzymatic approaches also reduce DNA degradation and sequencing errors, thereby increasing data quality in low‐abundance circulating tumour DNA (ctDNA) samples.

**FIGURE 2:**
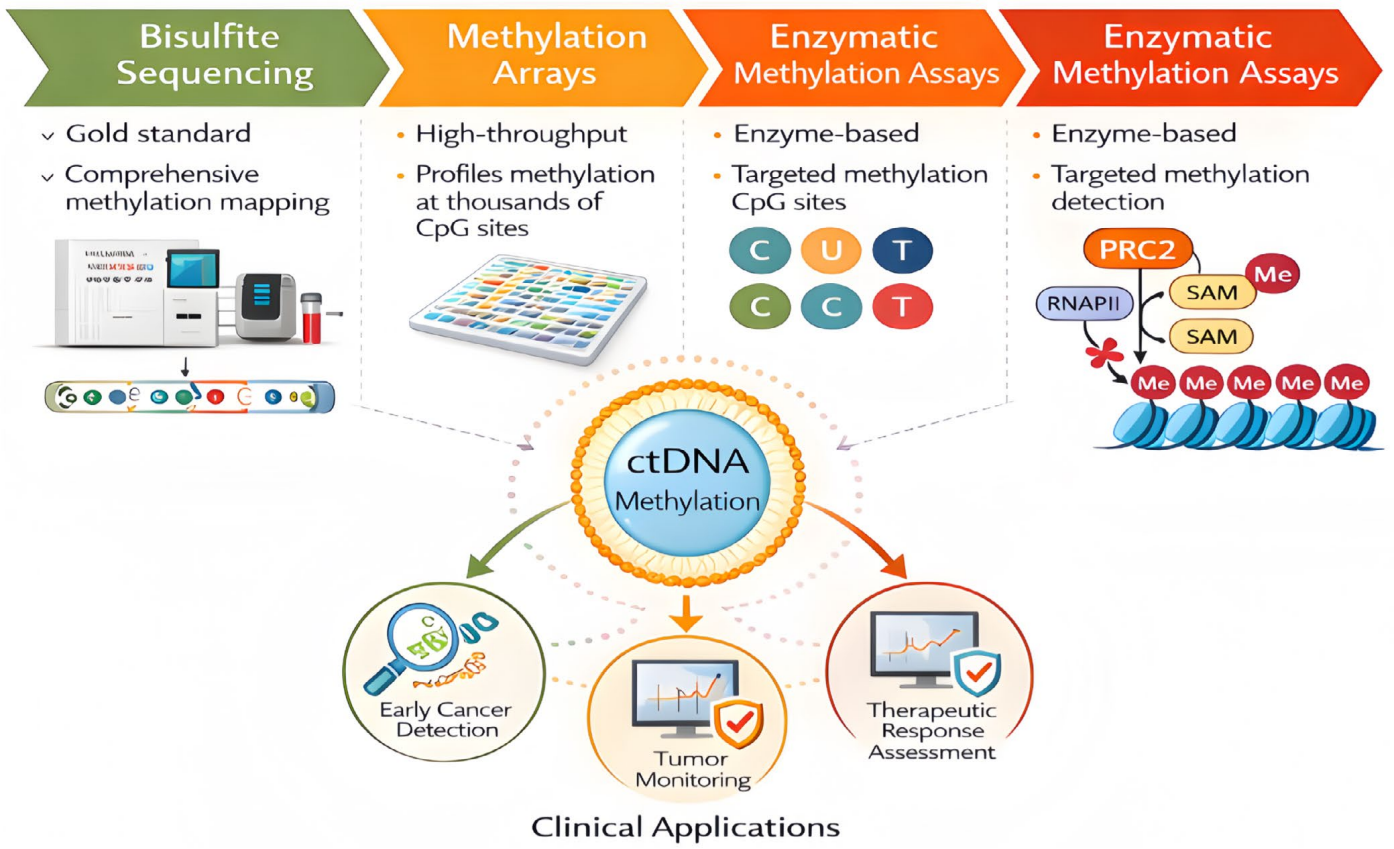
Technologies for epigenetic profiling of circulating tumour DNA (ctDNA) in cancer: comparative overview of bisulfite sequencing, methylation arrays, and enzymatic methylation assays with applications in early detection, tumour monitoring, and therapeutic response assessment.

Altogether, these technologies can provide a profile for early cancer diagnosis, control of tumour dynamics, and evaluation of therapeutic response using all non‐invasive ctDNA methylation patterns analysis. The selected approach is influenced by factors such as the intended clinical use, material availability, and resource capacity. A comprehensive summary of ctDNA epigenetic profiling technologies, principles, advantages, and clinical applications is presented in Figure 2.

### 5.2 Clinical Relevance in RCC Diagnosis, Prognosis, and MRD Monitoring

**FIGURE 3:**
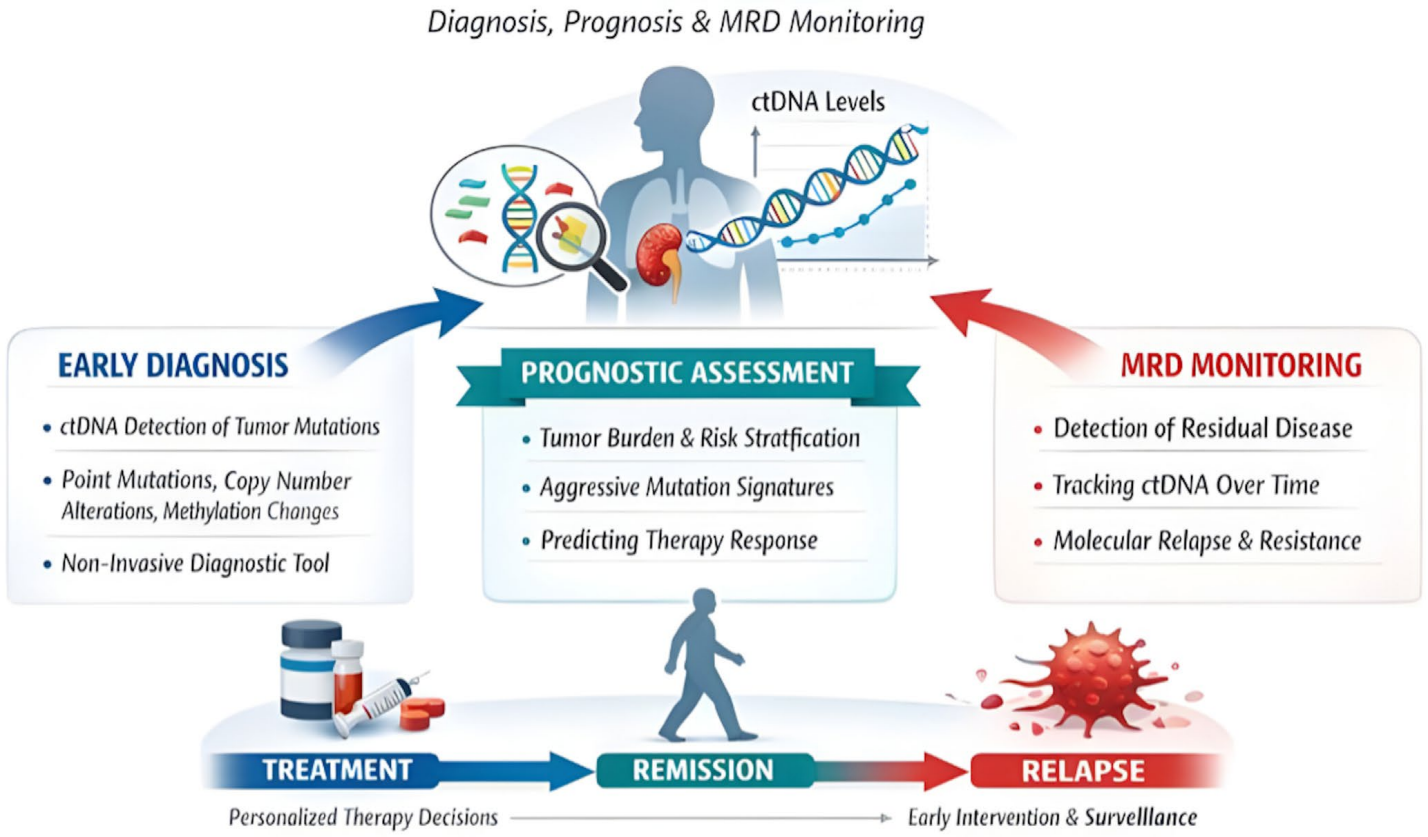
Clinical relevance of circulating tumour DNA (ctDNA) in RCC: integration of early diagnosis, prognostic assessment, and minimal residual disease (MRD) monitoring for personalised precision oncology and dynamic patient management.

Circulating tumour DNA (ctDNA) has emerged as a transformative biomarker in RCC, shedding light on disease biology and prognostication, and facilitating clinical decision‐making. Clinical applications range from early Diagnosis and prognosis evaluation to monitoring MRD, offering a non‐invasive approach compared to tissue biopsies. For Diagnosis, ctDNA enables detection of tumour‐specific genetic alterations (e.g., point mutations, copy number variations, and DNA methylation patterns), which are often difficult to detect with conventional imaging or biopsy methods [13]. This is particularly relevant for RCC, where lesions are often small, potentially heterogeneous, or occur in difficult‐to‐biopsy locations; therefore, a tissue‐based Diagnosis can be challenging [126]. Furthermore, ctDNA profiling enables serial sampling over time, allowing physicians to document the evolving molecular profile of a tumour and identify accruing changes that may be indicative of early oncogenic processes or therapeutic resistance. Major clinical applications of ctDNA in RCC: Diagnosis, prognosis, and MRD monitoring. The main clinical applications of ctDNA in RCC, focused on a chronological overview, are shown in Figure 3.

Prognostically, the amount of ctDNA and the detection of specific genetic or epigenetic variants have been found to correlate best with tumour burden, stage, and histopathological grade. Increased ctDNA levels are frequently associated with more advanced disease and inferior OS, whereas some mutation signatures might convey aggressive tumour behaviour as well as age response to therapy [127]. This knowledge can aid in risk stratification of patients and inform personalised treatment decisions, such as targeted therapies or immunotherapies. Longitudinal ctDNA monitoring also provides a sensitive means of tracking disease evolution. Through serial measurement of ctDNA levels, it is possible to prospectively identify individuals at high risk for disease progression or relapse before imaging or clinical evidence of disease progression is accrued.

ctDNA shows strong potential for minimal residual disease (MRD) monitoring in RCC by enabling sensitive detection of residual tumour cells after therapy. Rising ctDNA levels can indicate molecular relapse before radiographic evidence and reveal emerging resistance mutations, supporting earlier intervention and personalised treatment adjustment [127]. Overall, ctDNA offers a minimally invasive, multimodal approach for RCC management, enabling early detection, prognostication, and MRD monitoring while supporting personalised, evidence‐based clinical care.

#### 5.2.1 Critical Appraisal of Evidence and Clinical Readiness

While numerous studies highlight the promise of ctDNA fragmentomic and epigenetic biomarkers in RCC, clearer critical appraisal and contextualization enhance interpretability and translational relevance. Differentiating preclinical discoveries, retrospective validations, and prospective trials allows readers to assess the clinical readiness of these approaches. To summarise the current evidence, key clinical studies of ctDNA fragmentomic and epigenetic analyses in RCC are presented in Table 6, including study design, cohort characteristics, biomarker types, and main findings. This overview facilitates actionable insights and highlights areas requiring further prospective validation.

**TABLE 6:** Key clinical studies of ctDNA fragmentomic and epigenetic biomarkers in RCC.

Study (first author, year)	Study design	cohort/setting	Biomarker type	Key findings	Clinical implication
Yamamoto et al. [128] (cfDNA fragmentation)	Retrospective clinical study	53 clear cell RCC patients	ctDNA fragment size and mutation status	Shorter cfDNA fragment sizes correlated with presence of ctDNA and poor prognosis; ctDNA + fragmentation correlated with clinical course and survival	ctDNA fragmentomics correlates with disease monitoring and prognosis in RCC patients
Lasseter et al. [129] (cfMeDIP‐seq in mRCC)	Retrospective biomarker comparison	40 metastatic RCC patients (34 cfMeDIP‐seq)	cfMeDIP‐seq vs. variant analysis	cfMeDIP‐seq detected all mRCC cases (100% sensitivity, 88% specificity) versus lower sensitivity for variant only	cfMeDIP‐seq outperforms standard variant methods for detection in metastatic RCC
Nuzzo et al. [130] (cfMeDIP‐seq methylation in RCC)	Prospective/retrospective methylation study	69 RCC plasma +13 controls; urine cfDNA also examined	cfMeDIP‐seq methylomes	AUC ~0.99 for RCC plasma classification; AUC ~0.86 for urine cfDNA	High sensitivity/specificity methylation for early detection and classification
Smith et al. [2] (cfDNA genomic characterisation)	Retrospective genomic profiling	RCC plasma and urine cfDNA patients	cfDNA genomic and fragment characterisation	Comprehensive cfDNA profiles (fragmentation plus genomic alterations) distinguish renal tumours	Shows potential of multi‐feature cfDNA profiling including fragmentomics

### 5.3 Translational Integration of ctDNA Fragmentomic and Epigenetic Features in RCC

The ctDNA fragmentomic features and the epigenetic signatures represent complementary and biologically interconnected layers of tumour‐derived information that collectively enhance the translational utility of liquid biopsy in renal cell carcinoma (RCC). Fragment size distributions, end motifs, and nucleosome footprinting patterns reflect underlying chromatin organisation, nuclease activity, and transcriptional states within tumour cells, while alterations in DNA methylation and chromatin remodelling capture stable, tumour‐specific regulatory programs. In the context of RCC—a malignancy characterised by low levels of circulating tumour DNA—these orthogonal molecular signals provide a critical advantage over mutation‐only approaches by improving sensitivity and tumour specificity.

Integration of fragmentomic and epigenetic features enables a more comprehensive molecular portrait of RCC that directly informs clinical applications. Fragmentomics enhances the detectability of ctDNA by enriching tumour‐derived fragments and revealing tissue‐of‐origin signals. In contrast, epigenetic profiling contributes robust discrimination between malignant and non‐malignant cfDNA through RCC‐specific methylation patterns. When combined with integrative analytical frameworks, including machine‐learning‐based classifiers, these features significantly improve early cancer detection performance, particularly in localised disease where ctDNA abundance is minimal. This multi‐modal strategy addresses a significant limitation of current liquid biopsy approaches in RCC and provides a biologically informed basis for clinical translation.

**FIGURE 4:**
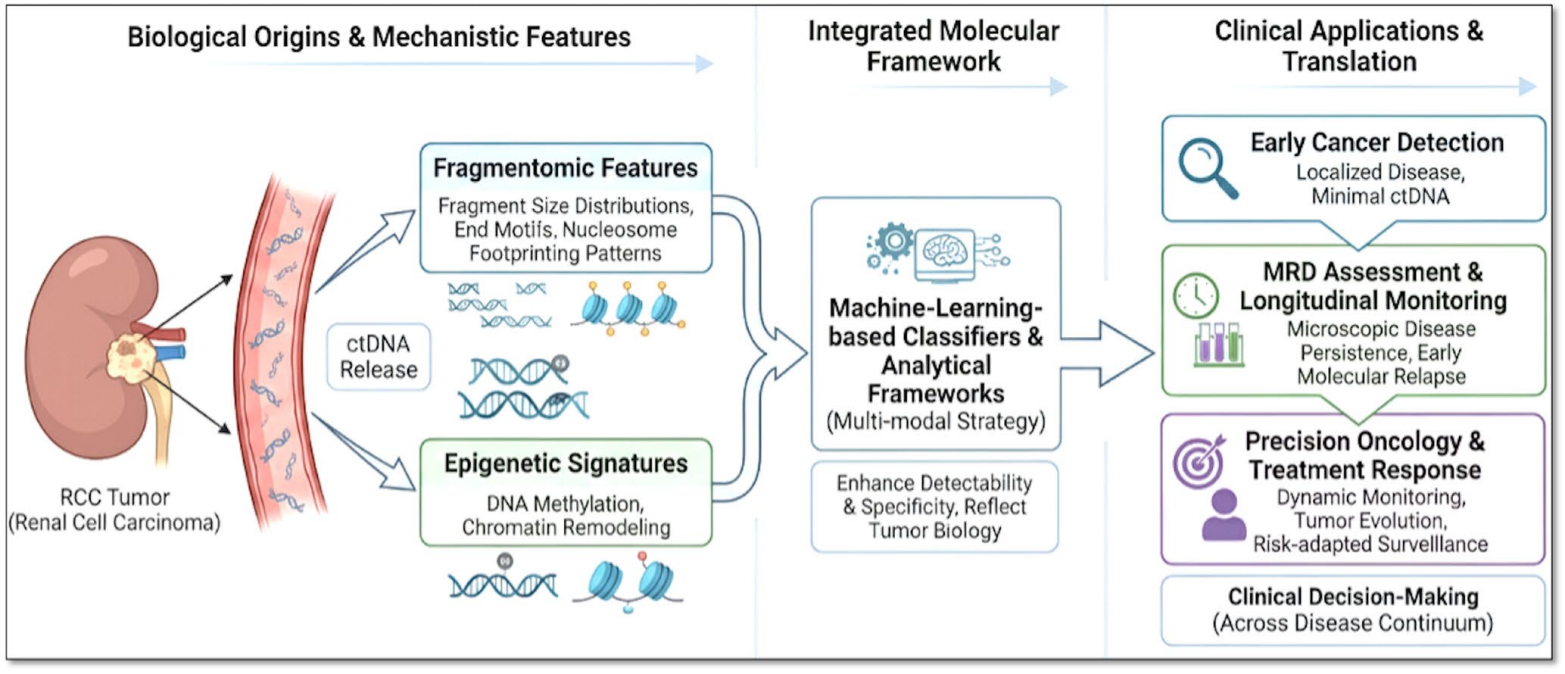
Translational continuum linking ctDNA fragmentomic and epigenetic mechanisms to clinical applications in renal cell carcinoma.

Beyond early detection, the integration of ctDNA fragmentomic and epigenetic features has significant implications for minimal residual disease (MRD) assessment and longitudinal monitoring in RCC, enabling highly sensitive detection of microscopic disease persistence and early molecular relapse in post‐surgical and post‐therapeutic settings. Fragmentation patterns reflecting nucleosome positioning and transcriptional activity, together with dynamic methylation changes, can reveal tumour‐derived signals months before radiographic recurrence, thereby supporting timely intervention, risk‐adapted surveillance, and treatment escalation. Moreover, longitudinal shifts in cfDNA fragmentation and epigenetic states provide insight into tumour burden, clonal evolution, and treatment response, complementing conventional metrics such as variant allele fraction and reinforcing the role of integrated ctDNA analysis in precision management of RCC, as seen in Figure 4.

## 6 Translational and Clinical Implications

### 6.1 Impact of Early RCC Detection on Clinical Outcomes

Earlier detection of RCC is associated with more favourable clinical outcomes owing to the possibility of timely intervention and broader therapeutic time windows. RCC is generally asymptomatic at the time of early diagnosis, and it is often discovered coincidentally or with advanced disease, which hampers treatment possibilities [131]. The timely detection of RCC enables curative treatments, including partial or radical nephrectomy, resulting in markedly improved overall survival compared to treatment for advanced disease. In addition, early recognition affords clinicians a broader treatment window for systemic therapies, such as targeted agents and immunotherapies, which are generally more efficacious in patients with a limited tumour burden and minimal metastatic dissemination. Longitudinal tracking of ctDNA can also play a role in postoperative surveillance to detect residual disease or early recurrence before radiological evidence appears, thereby enabling better management of patients with recurrent disease [127]. Ultimately, incorporating early detection approaches, such as ctDNA‐based assays, into RCC care pathways may lead to improved survival outcomes, reduced morbidity, and more individualised treatment.

### 6.2 Synergistic Benefits of Combined Fragmentomic and Epigenetic Analyses

Integrating ctDNA fragmentomic and epigenetic analysis represents a synergistic strategy that significantly increases the accuracy of diagnosing and stratifying risk in RCC. The Fragmentomic analysis considers characteristics such as ctDNA fragment size, end motif, and nucleosome positions, which can inform on the tissue of origin and tumour‐specific DNA shedding patterns [93]. Fragmentomics can also reveal patterns of apoptosis or necrosis, providing additional insights into tumour biology and aggressiveness. Concurrent with this, epigenetic profiling, most notably DNA methylation analysis, captures early tumour‐specific changes that take place during oncogenesis without any detectable mutations [13]. Methylation signatures that discriminate tumour subtypes are associated with patient outcome and have predictive value independent of histopathological features. Combining these complementary methods enables the identification of ctDNA with greater specificity and sensitivity, particularly in early‐stage tumours where plasma ctDNA concentrations are low and traditional biomarkers may be inadequate.

**FIGURE 5:**
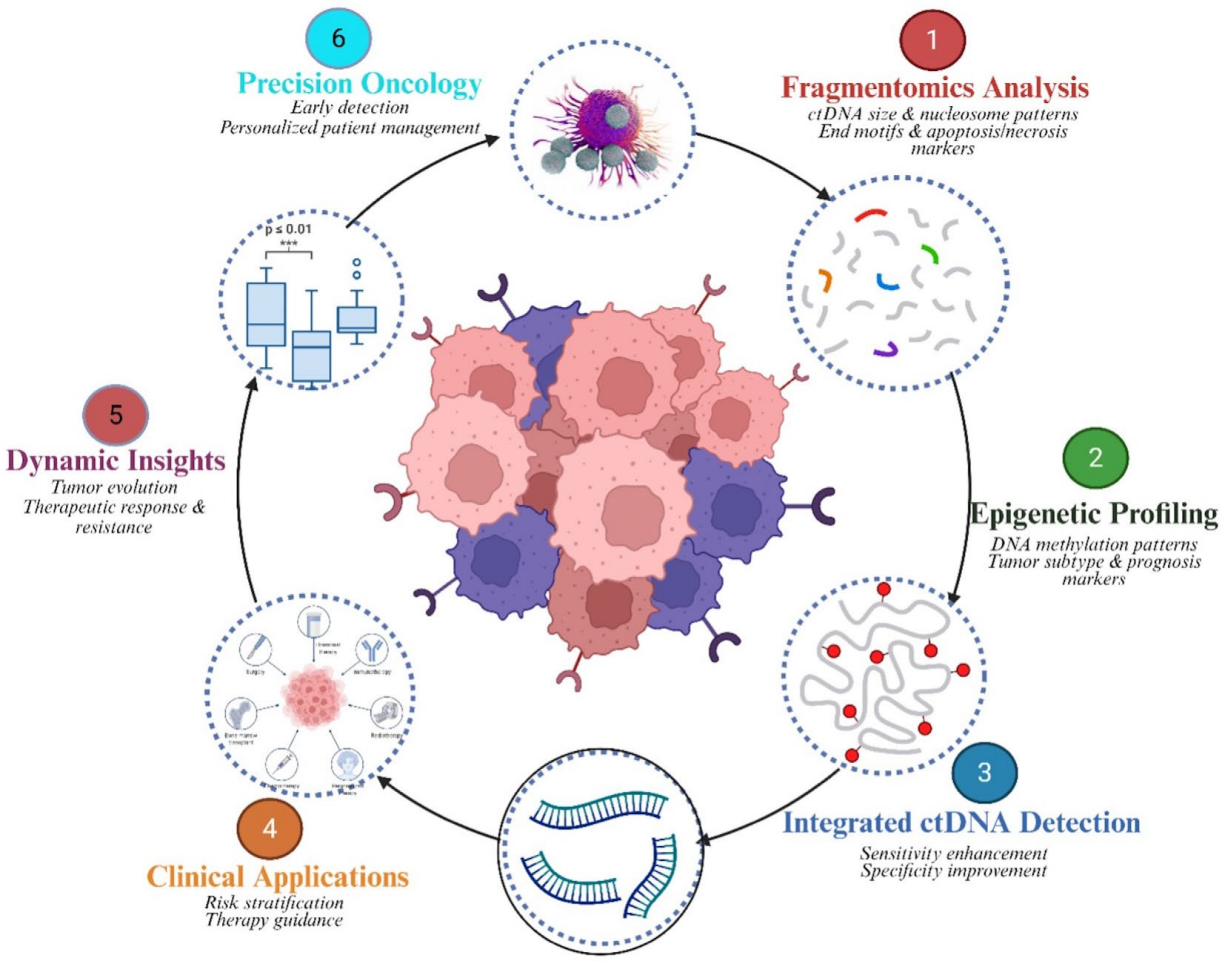
Synergistic benefits of combined ctDNA fragmentomic and epigenetic analyses in RCC: enhanced diagnostic accuracy, risk stratification, tumour evolution monitoring, and personalised precision oncology applications.

Several studies demonstrate that integrating ctDNA fragmentomic and methylation analyses outperforms single‐modality approaches for cancer detection and discrimination from non‐cancer [19]. This combined strategy enhances risk stratification in RCC, supports early identification of recurrence and treatment response, and enables longitudinal monitoring of tumour evolution and emerging therapy resistance [132]. Collectively, the integration of ctDNA fragmentomic and epigenetic studies represents an innovative approach to RCC diagnostics and prognostics. Through the complementary benefits of these modalities, clinicians can enable earlier detection, more accurate risk assessment, and improved patient stratification, driving better clinical outcomes and personalised oncology care. The collaborative functions, clinical potential implications, and concerted advantages of integrated fragmentomic and epigenetic analyses are summarised in Figure 5.

### 6.3 Clinical Trials and Studies Supporting Combined Biomarker Use

Since then, numerous clinical trials and studies have investigated the use of combined ctDNA fragmentomics and epigenetic analysis in early detection and monitoring of RCC. These methods leverage the characteristic fragmentation patterns and methylation signatures of ctDNA to enhance diagnostic sensitivity and risk stratification. For example, the DECIPHER‐RCC study developed a cfDNA fragmentomics‐based liquid biopsy for noninvasive RCC detection, showing that ctDNA fragmentation patterns provide valuable information on tumour presence and characteristics, supporting its potential as a diagnostic tool [76].

Another vital trial is the study of the predictive value of cfDNA size profiles in patients with advanced carcinoma treated with immune checkpoint inhibitors. As the trial had already reported that specific sizes of cfDNA fragments were correlated with PFS, this indicates that ctDNA fragment distribution patterns could be used as a “biomarker” for response to treatment and cancer progression [133]. These findings highlight the utility of integrating ctDNA fragmentomics and epigenetic profiling in clinical practice. By combining these biomarkers, clinicians can achieve more precise early diagnosis and risk stratification, as well as individualise treatment options for patients with RCC.

### 6.4 Future Perspectives in Clinical Implementation

This integration of ctDNA fragmentomics and epigenetic profiling into routine RCC screening would be a game‐changing step toward non‐invasive, early‐detection methods. Present clinical approaches largely rely on imaging modalities and tissue biopsies, which sometimes fail to detect tumours at early stages or are contraindicated in some patients. By way of contrast, liquid biopsy methods (specifically analyses that focus on ctDNA fragmentation patterns and methylation signatures) offer a less invasive strategy with the possibility to detect RCC in an earlier and more accurate manner [76].

Improvements in ctDNA fragmentomics have demonstrated that characteristics such as nucleosome positioning, fragment length, and end motifs can provide insights into tumour presence and origin. For example, the ctDNA of RCC patients often exhibits a distinct size distribution pattern compared to that of cfDNA from healthy donors, providing a means to distinguish between malignant and benign tumours. At the same time, epigenetic change, especially abnormal DNA methylation changes, appeared at an early stage in the pathogenesis of RCC, and these changes have been observed in ctDNA, through which it may serve as a molecular marker for diagnosis and risk stratification [13].

The translation of these technologies to clinical practice will necessitate the establishment of universal protocols for the collection, processing, and analysis of ctDNA to ensure reproducibility and reliability. Large‐scale validation studies are necessary to determine the test's sensitivity, specificity, and clinical utility across various populations. A combined effort among research institutions, healthcare providers, and regulatory agencies will be essential to translate these assays into routine clinical use. Incorporation of ctDNA fragmentomics and epigenetic analysis into RCC surveillance programs offers significant potential for early detection, improved prognosis, and personalised therapeutic guidance. Further studies, clinical validation, and advancements in technology are required to fully utilise these non‐invasive markers to their maximum potential in RCC management [7].

## 7 Minimal Residual Disease (MRD) Assessment and Precision Monitoring

### 7.1 Clinical Significance of MRD in RCC

RCC minimal residual disease (MRD) is comprised of cancer cells left in your body after you have received curative‐intent treatment, such as surgery or systemic therapy, that cannot be seen on imaging tests or other analyses checked by your doctor, but may later cause recurrence. These remaining microscopic cells can serve as a pool for tumour recurrence or metastasis, rendering MRD a significant prognostic parameter in RCC. The detection of MRD is closely associated with an increased risk of relapse and poorer overall survival; meanwhile, its absence is linked to longer disease‐free survival [134].

From a clinical perspective, MRD kinetics can guide treatment personalization. Patients with measurable MRD may profit from adjuvant systemic therapy, closer follow‐up, or inclusion into clinical trials for eradication of remaining disease. In contrast, MRD‐negative patients can be spared unnecessary treatments and toxicities [135]. The MRD status also contributes to the division of patients into risk groups, which guides clinicians in deciding who should receive intensive surveillance and whom they should intervene for earlier.

**FIGURE 6:**
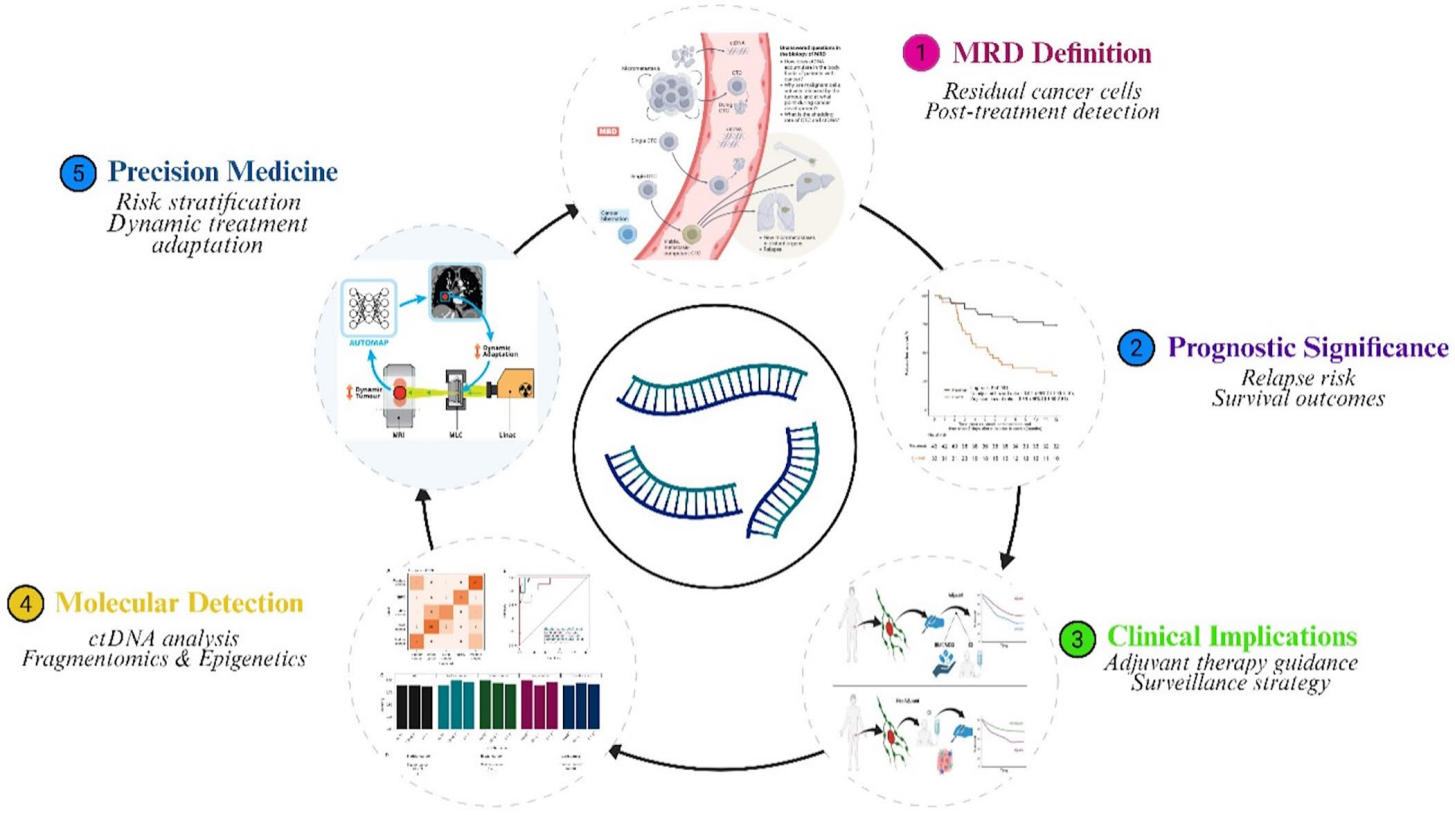
Comprehensive overview of MRD in RCC: definition, prognostic significance, clinical implications, and molecular detection using ctDNA, fragmentomics, and epigenetic profiling for precision medicine approaches.

In recent years, Contrast‐enhanced (CE) imaging, alongside advancements in ctDNA analysis, has offered complementary approaches for the detection, characterisation, and monitoring of RCC. Epigenetic profiling has made significant strides in enhancing MRD detection by providing ultra‐sensitive and specific molecular tools for detecting RCC in blood at levels below clinical or radiologic evidence of disease recurrence. These molecular strategies can provide real‐time awareness of residual disease, facilitate early detection of relapse, and enable dynamic analysis of treatment response, thereby facilitating the successful application of precision medicine in RCC. The specific definition, clinical significance, and implications of MRD for the prognosis and therapeutic decision‐making in RCC are illustrated in Figure 6.

### 7.2 Utility of ctDNA Fragmentomics and Epigenetics in MRD Detection

**FIGURE 7:**
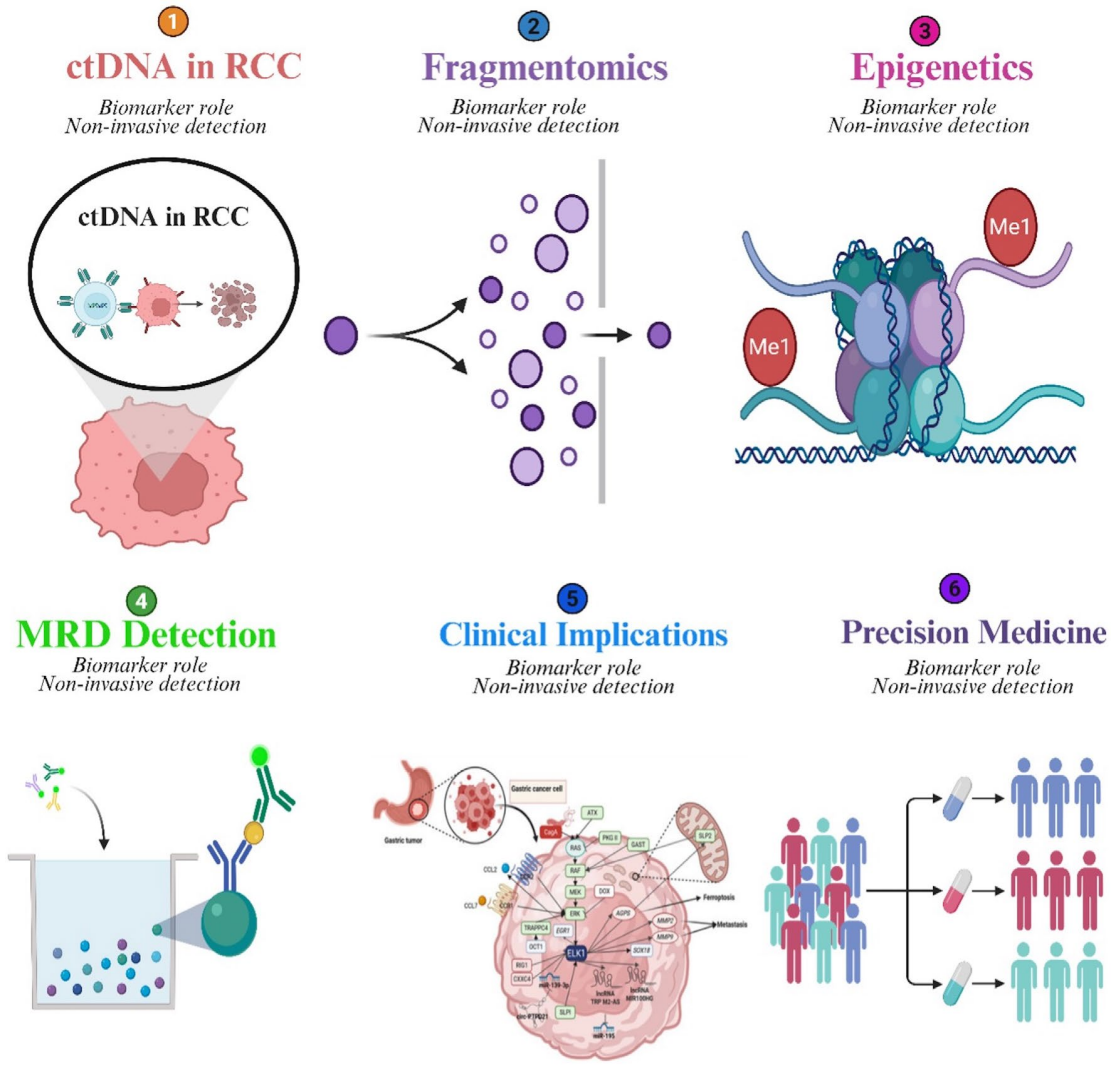
Comprehensive overview of circulating tumour DNA (ctDNA) fragmentomics and epigenetic profiling in minimal residual disease (MRD) detection for renal cell carcinoma (RCC): integration of ctDNA biomarker roles, fragmentomic features, epigenetic modifications, early MRD detection, clinical implications, and precision medicine approaches.

Circulating tumour DNA (ctDNA) fragmentomics and epigenetic profiles have recently been established as sensitive tools for MRD detection in RCC. Microscopic residual tumour cells cannot always be detected by conventional imaging and histopathology, which may lead to missed opportunities for timely treatment and recurrent disease [136]. ctDNA analysis detects tumour‐derived fragments in blood, and fragmentomics leverages size, nucleosome, and end‐motif patterns to enhance the detection of low‐abundance tumour DNA [93]. Combining fragmentomics with cancer‐specific DNA methylation enhances MRD detection, with fragmentomics providing sensitivity and methylation profiling providing specificity, enabling earlier intervention than conventional methods [132]. The clinical relevance of ctDNA‐based MRD detection is crucial. By detecting patients with molecular residual disease, adjuvant treatments can be individualised, and monitoring intensified, potentially leading to a survival benefit. Indeed, serial ctDNA monitoring enables the real‐time evaluation of treatment response and the early detection of disease relapse, thereby providing the basis for precision medicine in RCC. The full roles and number of ctDNA fragmentomics and epigenetic signatures in MRD detection are detailed in Figure 7.

### 7.3 Role in Treatment Response Monitoring and Early Relapse Detection

Circulating tumour DNA (ctDNA) fragmentomics and epigenetic profiling play crucial roles in tracking treatment responses and detecting early relapses for RCC. Conventional imaging methods often provide late or indirect evidence of therapeutic response, whereas ctDNA can offer real‐time molecular evaluation of tumour kinetics. Quantitative variations in ctDNA levels, fragmentomic features, or methylation patterns reflect tumour burden and treatment response, providing clinicians with near‐real‐time monitoring of the effectiveness of surgical, targeted therapy, or immunotherapy. Dynamic tracking of ctDNA may identify MRD or molecular‐based relapse earlier than radiological and clinical progression. Increasing ctDNA levels, as well as the reappearance of methylated tumour‐specific signatures, could signal early recurrence, providing an opportunity for intervention [132]. This provides an opportunity for clinicians to modify treatment strategies (e.g., escalating systemic therapy, changing targeted agents, or participating in clinical trials) based on molecular evidence rather than waiting until overt clinical progression occurs [19].

**FIGURE 8:**
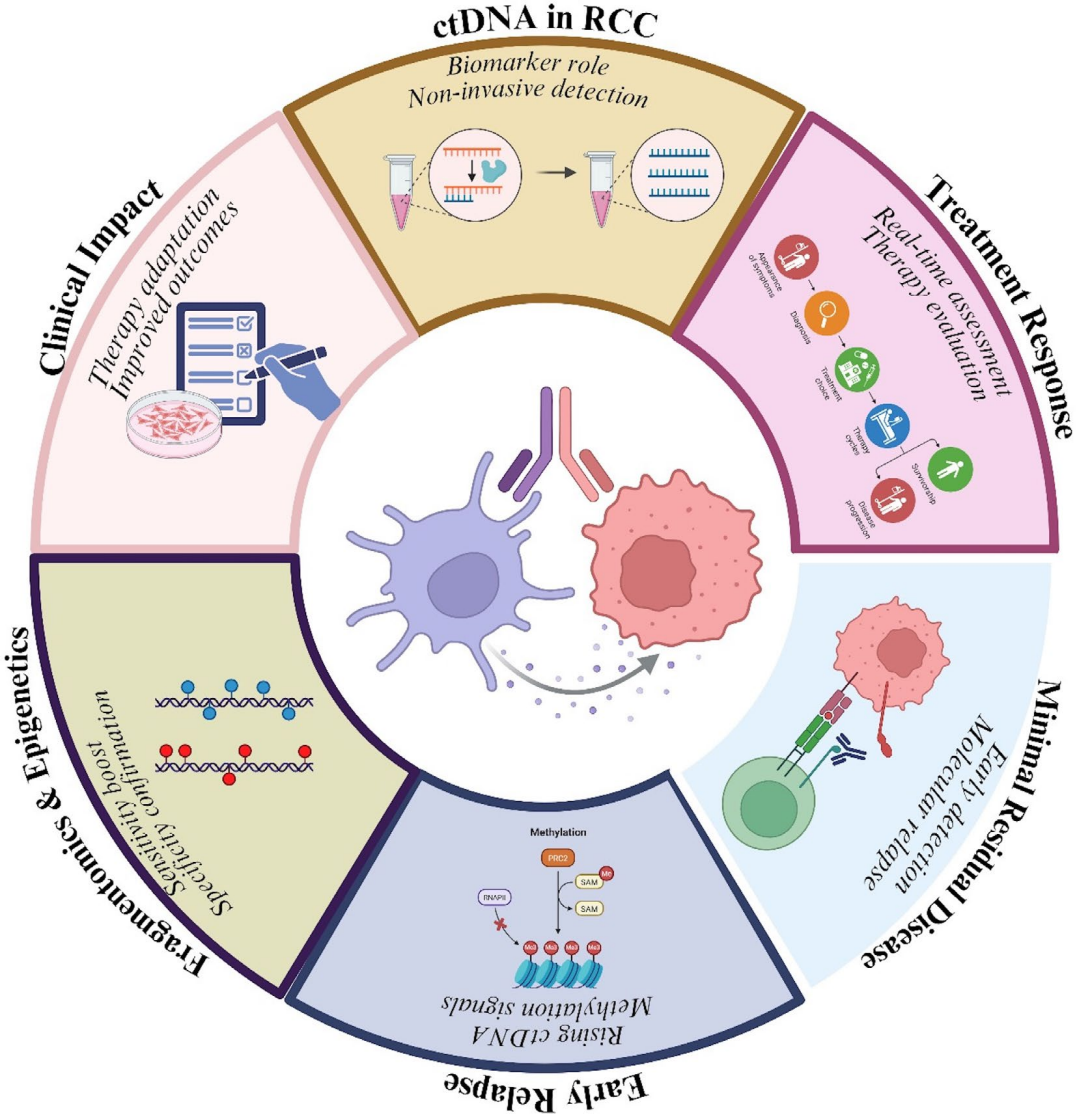
Role of circulating tumour DNA (ctDNA) fragmentomics and epigenetic profiling in monitoring treatment response and early relapse detection in renal cell carcinoma (RCC): integration of real‐time assessment, minimal residual disease detection, and precision oncology approaches.

Moreover, the incorporation of fragmentomic and epigenetic analyses increases sensitivity and specificity for monitoring ctDNA. Fragmentomics can detect subtle ctDNA changes that reflect residual disease. Seeking tumour‐specific evidence in methylation signals will help to confirm and reduce potential false positives (FP) and therefore enhance confidence in clinical decision‐making. The longitudinal assessment of these molecular markers can also help better understand tumour evolution and the selection of therapy‐resistant clones, thereby directing precision oncology strategies in renal cell carcinoma. ctDNA‐based monitoring is a non‐invasive dynamic test that may complement conventional imaging and histopathology. It enables early relapse detection and real‐time assessment of therapeutic response, facilitating treatment guidance tailored to individual patients and thereby improving patient care, potentially enhancing survival outcomes in RCC [127]. The specific roles, sub‐components, and clinical implications of ctDNA monitoring are presented in Figure 8.

### 7.4 Case Studies and Ongoing Research

The clinical utility of ctDNA for monitoring MRD in RCC is supported by several case reports and trial studies. For example, at the 2024 American Society of Clinical Oncology (ASCO) meeting, a study demonstrated the use of the Signatera ctDNA assay to monitor MRD in patients with RCC. This tumour‐informed, individualised strategy showed high concordance with gold‐standard imaging, suggesting its potential as a non‐invasive tool for early detection of relapse and tracking of treatment.

In another study, Peng et al. [7] established a cell‐free DNA fragmentomics‐based liquid biopsy for noninvasive diagnosis of RCC. This approach exhibited increased detection sensitivity for microscopic RD and can be used as a suitable substitute to the conventional biopsy techniques. Additionally, Lyskjær et al. [137] presented in the literature on ctDNA among patients with RCC, highlighting how it could be used to determine treatment response and early detection of relapse.

Further investigation is underway to evaluate the role of ctDNA in the management of RCC. For instance, the DIRECT study (a prospective, multisite trial) is evaluating the feasibility and clinical relevance of ctDNA in 188 patients with aggressive RCC. Initial reports suggest a correlation between ctDNA dynamics and clinical response, indicating the potential for integrating this information into patient‐specific treatment algorithms.

These case reports and studies support the increasing evidence for clinically actionable and robust ctDNA‐based MRD detection in RCC. With further technological development and more extensive validation through prospective clinical trials, ctDNA assays may play a crucial role in RCC management, potentially providing an effective, non‐invasive tool for early diagnosis and postoperative monitoring of minimal residual disease.

## 8 Challenges and Limitations

### 8.1 Technical Barriers to Clinical Application

Circulating tumour DNA (ctDNA) assays in RCC are hindered by numerous technical challenges, leading to reduced sensitivity, specificity, and reproducibility. Sensitivity is a significant issue, given that ctDNA is often present at low proportions in total cell‐free DNA, particularly in early‐stage or minimal residual disease, which increases the sensitivity of matches and makes the challenging detection of tumoral DNA at low abundance even more difficult. Similarly, specificity may be influenced by non‐tumour‐specific DNA alterations, such as clonal haematopoiesis, which can lead to false positives if not appropriately corrected for [138].

Assay standardisation and reproducibility are also obstacles. Pre‐analytical biases in ctDNA yield and quality may arise from variability in blood sample collection, processing, and storage. Variations in sequencing platforms, library sample preparation, or bioinformatic pipelines can also cause discordant results between laboratories or studies [132]. This highlights the importance of stringent methodologies (in terms of protocol harmonisation, validation, and quality assurance) for ctDNA‐based assays to yield consistent and clinically relevant results.

Efforts to address these challenges include the development of highly sensitive assays (digital droplet PCR, ultra‐deep next‐generation sequencing) and the combination of fragment‐based and methylation‐based analyses to enhance the sensitivity of detection for low‐abundance ctDNA [132]. Moreover, harmonisation of pre‐analytical workflows, standard reference materials, and cross‐laboratory benchmarking are emerging as critical solutions for improving reproducibility and clinical adoption.

### 8.2 Biological Complexities Impacting ctDNA Analysis

Biological diversity poses a challenge for the accurate analysis and interpretation of RCC ctDNA. Tumour heterogeneity, which refers to genetic, epigenetic, and phenotypic differences not only between but also within tumours, is one of these critical determinants. Various mutations or methylation patterns may exist in different areas of the cancer, resulting in heterogeneous circulating tumour DNA (ctDNA) profiles within the blood [139]. This heterogeneity underscores the need for multi‐omics approaches that integrate fragmentomics, methylation profiling, and genomic analysis to comprehensively capture tumour signals.

Shedding variability in ctDNA is another crucial consideration. The amount of ctDNA, which varies among patients and tumour types, as well as across disease stages, can be released into the circulation. Early tumours or micrometastases can shed tiny amounts of ctDNA, making the detection of ctDNA difficult, whereas large and more aggressive tumours may release greater levels of DNA [140]. Factors such as tumour vascularization, cellular death mechanisms, and DNA clearance rates contribute to inter‐ and intra‐patient variability. Longitudinal ctDNA sampling combined with integrative multi‐omics profiling can mitigate these biological confounders by providing dynamic monitoring of tumour evolution, treatment response, and minimal residual disease [141].

### 8.3 Practical and Clinical Translation Challenges and Strategies to Overcome

The clinical translation of ctDNA analysis in RCC faces several practical and systemic hurdles. High‐sensitivity assays, such as ultra‐deep sequencing, fragmentomics, or methylation profiling, require substantial resources and specialised infrastructure, making these tests cost‐prohibitive. Accessibility is limited because ctDNA analysis is currently feasible mainly at specialised centers, thereby restricting patient access in resource‐constrained regions. In addition, extensive analytical and clinical validation is required to meet regulatory standards for sensitivity, specificity, and reproducibility. At the same time, the lack of universal guidelines—including detection thresholds and standardised practice criteria—further complicates inter‐study comparisons and integration into routine care. To overcome these hurdles, strategies include scaling laboratory infrastructure, reducing assay costs, standardising protocols, and generating robust prospective clinical evidence to support routine clinical integration.

Several actionable strategies have been proposed to address these challenges. Multi‐omics integration, combining fragmentomics, epigenetics, and genomics, improves sensitivity and specificity, particularly in low‐abundance ctDNA, by leveraging fragment size distribution, nucleosome mapping, and tumour‐specific methylation patterns to reduce false positives and capture heterogeneous tumour signal [19]. Longitudinal sampling enables dynamic monitoring of tumour burden, treatment response, and minimal residual disease, overcoming the limitations of single‐point measurements. Assay optimization, including ultrasensitive sequencing methods, digital PCR, and harmonised pre‐analytical workflows, enhances reproducibility and clinical relevance [138]. Furthermore, the validation of these assays in an extensive, prospective clinical study will provide evidence for regulatory approval and implementation into clinical practice.

Moreover, AI‐driven approaches are emerging to integrate multi‐omics data, identify subtle molecular patterns, and improve predictive accuracy. Finally, prospective, multicenter clinical validation is essential to provide evidence for regulatory approval and facilitate adoption into routine clinical practice. Collectively, these strategies—including multi‐omics integration, AI‐driven analysis, harmonised workflows, longitudinal sampling, and prospective validation—represent a comprehensive framework to transform ctDNA analysis into a robust and clinically actionable tool for early detection, MRD monitoring, and treatment response assessment in RCC.

## 9 Conclusion

The burgeoning field of liquid biopsy is poised to revolutionise the management paradigm of RCC, moving beyond the constraints of traditional imaging and invasive tissue biopsies. This review has synthesised the compelling evidence for two particularly promising analytical domains: ctDNA fragmentomics and epigenetic signatures. Individually, each approach addresses a critical weakness of mutation‐based ctDNA assays in RCC. Fragmentomics leverages the inherent physical and structural characteristics of tumour‐derived DNA, such as shorter fragment lengths and distinct nucleosome positioning, to achieve enhanced detection sensitivity, especially in early‐stage and low‐shedding tumours where mutant allele fractions are minimal. Epigenetic profiling, particularly of DNA methylation, captures tissue‐ and cancer‐specific alterations that occur early in tumorigenesis, providing a robust and specific signal for distinguishing RCC from benign masses, determining histological subtypes, and identifying aggressive variants such as sarcomatoid differentiation. Integrating fragmentomic, epigenetic, and genomic data provides a comprehensive tumour snapshot, with assays such as DECIPHER‐RCC achieving high diagnostic accuracy for the noninvasive detection of small renal masses. Combined biomarkers enable early MRD detection and precision monitoring, allowing timely intervention, guiding adjuvant therapy, and tracking clonal evolution and treatment resistance for personalised care. Despite their promise, clinical translation of ctDNA faces technical, biological, and practical challenges, including assay sensitivity, standardisation, tumour heterogeneity, patient variability, cost, accessibility, and the need for large‐scale validation. Future work should address these challenges via technological improvements, consensus guidelines, and clinical trials. Integrating ctDNA fragmentomics and epigenetics offers a precise, non‐invasive approach for RCC, enabling early detection, risk stratification, and personalised monitoring.

## Author Contributions

**Hossam Kamli:** conceptualization, investigation, writing – original draft, writing – review and editing, validation, methodology. **Najeeb Ullah Khan:** conceptualization, investigation, validation, visualization, writing – review and editing, writing – original draft.

## Funding

The authors have nothing to report.

## Ethics Statement

The authors have nothing to report.

## Consent

All the authors have read and approved the article for publication.

## Conflicts of Interest

The authors declare no conflicts of interest.

## Data Availability

The data that support the findings of this study are available from the corresponding author upon reasonable request.
